# Elucidation of Physiological, Transcriptomic and Metabolomic Salinity Response Mechanisms in *Medicago sativa*

**DOI:** 10.3390/plants12102059

**Published:** 2023-05-22

**Authors:** Stacy D. Singer, Madeline Lehmann, Zixuan Zhang, Udaya Subedi, Kimberley Burton Hughes, Nathaniel Z.-L. Lim, Rodrigo Ortega Polo, Guanqun Chen, Surya Acharya, Abdelali Hannoufa, Tao Huan

**Affiliations:** 1 Lethbridge Research and Development Centre, Agriculture and Agri-Food Canada, Lethbridge, AB T1J 4B1, Canada; 2 Department of Agricultural, Food and Nutritional Science, University of Alberta, Edmonton, AB T6G 2P5, Canada; 3 Department of Chemistry, University of British Columbia, Vancouver, BC V6T 1Z1, Canada; 4 London Research and Development Centre, Agriculture and Agri-Food Canada, London, ON N5V 4T3, Canada

**Keywords:** alfalfa, forage, gene expression, metabolites, salinity stress response

## Abstract

Alfalfa (*Medicago sativa* L.) is a widely grown perennial leguminous forage crop with a number of positive attributes. However, despite its moderate ability to tolerate saline soils, which are increasing in prevalence worldwide, it suffers considerable yield declines under these growth conditions. While a general framework of the cascade of events involved in plant salinity response has been unraveled in recent years, many gaps remain in our understanding of the precise molecular mechanisms involved in this process, particularly in non-model yet economically important species such as alfalfa. Therefore, as a means of further elucidating salinity response mechanisms in this species, we carried out in-depth physiological assessments of *M. sativa* cv. Beaver, as well as transcriptomic and untargeted metabolomic evaluations of leaf tissues, following extended exposure to salinity (grown for 3–4 weeks under saline treatment) and control conditions. In addition to the substantial growth and photosynthetic reductions observed under salinity treatment, we identified 1233 significant differentially expressed genes between growth conditions, as well as 60 annotated differentially accumulated metabolites. Taken together, our results suggest that changes to cell membranes and walls, cuticular and/or epicuticular waxes, osmoprotectant levels, antioxidant-related metabolic pathways, and the expression of genes encoding ion transporters, protective proteins, and transcription factors are likely involved in alfalfa’s salinity response process. Although some of these alterations may contribute to alfalfa’s modest salinity resilience, it is feasible that several may be disadvantageous in this context and could therefore provide valuable targets for the further improvement of tolerance to this stress in the future.

## 1. Introduction

Alfalfa (*Medicago sativa* L.) is an extensively grown perennial forage legume that is used for seed production, dehydrated products, and ruminant feed in the form of hay, silage, and pasture [1,2]. The popularity of this forage crop stems from its numerous benefits, including its symbiotic relationship with rhizobia and consequent ability to fix atmospheric nitrogen, which allows for its low input status and the enhancement of soil fertility. In addition, alfalfa also provides relatively high yields, nutritive value, tolerance of cutting/grazing, palatability, and environmental adaptability [3], which also contribute to its widespread use.

Soil salinity is an issue that affects arable land globally, with the highest incidence in semi-arid and arid regions [4]. In irrigated areas, salinity-induced land degradation has been suggested to be responsible for losses of approximately USD 27.3 billion in agricultural production per annum [5], and this is projected to worsen in coming years due to climate change effects and changing agricultural practices. For example, climate change-derived rises in sea levels, heavy precipitation, and flooding can lead to escalating salinization of groundwater in coastal regions [6]. Furthermore, increasing frequencies and intensities of droughts will decrease water availability and, at the same time, lead to an expanded need for irrigation [6], which can exacerbate groundwater decline and increase salinization [7].

Since alfalfa has sizeable water requirements, a large proportion of producers in major growing regions make use of irrigation [8], which means that increasing levels of soil salinization could be particularly problematic for this crop. Although alfalfa typically exhibits moderate levels of salinity tolerance [1], its biomass production can still be severely impacted by this type of stress, and it is one of the principal environmental issues limiting its production [9]. This, together with the prediction that the production of ruminant-derived goods will need to be augmented by approximately 88% by the year 2050 [10], indicates that there is a clear need for the development of novel cultivars with improved salinity resilience in coming years.

When plants are exposed to saline growth conditions, they characteristically suffer from osmotic stress resulting from low water availability, as well as mineral imbalances, ion toxicity, and oxidative stress resulting from the buildup of reactive oxygen species (ROS) [11]. This, in turn, negatively affects numerous traits in alfalfa, including biomass yield, nitrogen fixation capacity, and seed germination [9,12,13]. To better cope with salt in their environment, plants have evolved response strategies that typically fall into three broad categories, including the exclusion of salt ions via the modulation of transporter activity, the compartmentalization of sodium ions into vacuoles via the action of tonoplast Na^+^/H^+^ exchangers, and the adjustment of osmotic and oxidative stress response mechanisms [14]. These responses involve various physiological, biochemical, and molecular alterations that are generally initiated through the modulation of signal transduction components such as Ca^2+^, ROS, and phytohormones [15]. These signals alter the expression of a variety of genes encoding transcription factors, including WRKY, AP2/ERF, MYB, and bZIP family members [16], and lead to numerous downstream effects, including stomatal closure, activation of the SALT OVERLY SENSITIVE (SOS) pathway and subsequent exclusion of Na^+^, enhancement of the activities/levels of various enzymatic and non-enzymatic antioxidants, and an increase in the quantity of compatible solutes [17,18,19,20].

Although a wealth of information has been accrued over recent years in the context of salinity response mechanisms in plants, gaps remain in our understanding of this process, especially in non-model crops such as alfalfa. Transcriptomic approaches have become a popular method for examining complex salinity response mechanisms in various plant species, including alfalfa [21,22,23,24,25,26,27,28,29]; however, the vast majority of these studies have focused on relatively short salinity treatment durations (hours to one week) and/or root tissues [21,22,24,25,26,27,28,29]. While these studies have provided considerable insight into responses related to the first phase of salinity response, where roots are the first point of exposure, far less is known regarding longer-term responses in aboveground tissues, which are also known to provide important adaptive mechanisms for withstanding this type of stress [30]. Furthermore, although metabolomic analyses are beginning to contribute to the unraveling of salinity response in other plant species in recent years [31,32,33], only a very small number of metabolomic assessments of alfalfa have been conducted under salinity stress to date, and these were limited to metabolites targeted to a specific selection of pathways [34,35] or lipidomic profiles [36]. Therefore, as a means of furthering our understanding of salinity response mechanisms in alfalfa, we sought to assess the physiological responses of *M. sativa* cv. Beaver, as well as transcriptomic and untargeted metabolomic changes in leaf tissues, following extended exposure to salinity (NaCl), when plants were likely suffering from both osmotic and ionic stress. Such data will help unravel the complex response mechanisms of this important forage species under salt stress, and will aid in the identification of putative genes, metabolites, and pathways involved in this process to facilitate future breeding endeavors.

## 2. Results

### 2.1. Salinity-Treated Alfalfa Plants Exhibited Reductions in Aboveground Growth and Water Content Compared to Control-Treated Plants

**Figure 1:**
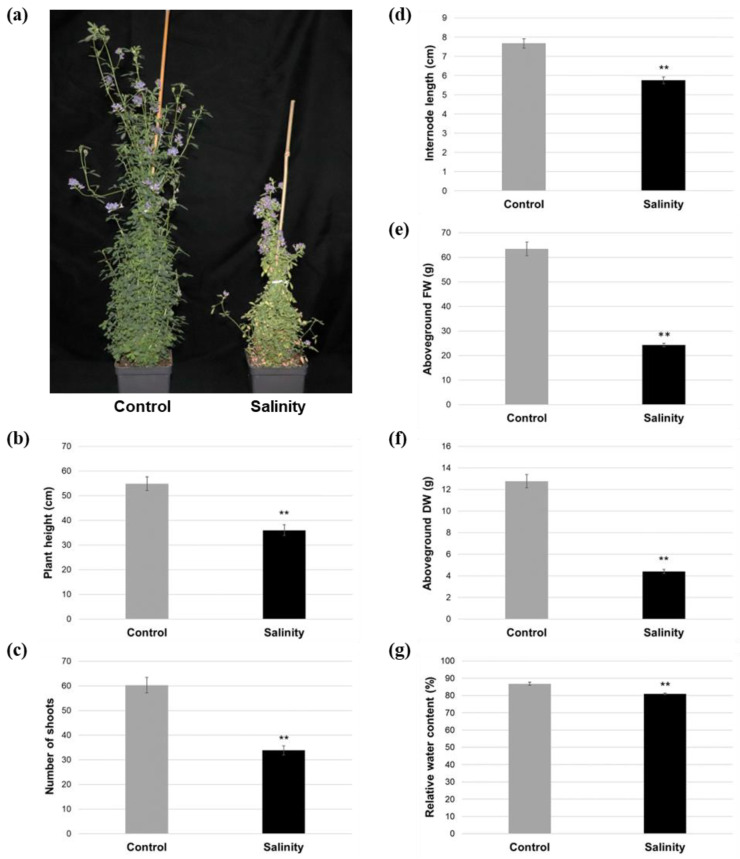
Aboveground morphology of alfalfa cv. Beaver under control and saline conditions. (**a**) Representative images taken 30 days after the initiation of treatment. (**b**–**f**) Length of the longest shoot, number of total shoots, internode length, and aboveground fresh weight (FW) and dry weight (DW) under control and saline conditions. Measurements were taken 21 (**b**–**d**) or 31 (**e**,**f**) days following the initiation of treatment. (**g**) Leaf relative water contents 21 days following the initiation of treatment. For all graphs, blocks represent the mean value of 10 biological replicate plants derived from vegetative stem cuttings (**b**–**f**) or 2 leaves from each of 5 biological replicate plants (**g**) for each treatment, and bars denote standard errors. Asterisks indicate a statistically significant difference between salinity and control treatments in each graph (**, *p* ≤ 0.01).

To identify morphological changes in alfalfa cv. Beaver that were associated with salinity stress, control- and salinity-treated plants were evaluated for various morphological parameters. By approximately 3 weeks following the initiation of treatment, salinity-treated plants were substantially smaller with senescing leaves compared to control-treated plants (Figure 1a), and they exhibited significant decreases in plant height (34.29% relative reduction), total number of shoots (43.95% relative reduction), internode length (24.97% relative reduction), and aboveground fresh weight (FW; 61.74% relative reduction) and dry weight (DW; 65.43% relative reduction; Figure 1b–f). Similarly, salinity-treated plants also displayed a significant 6.62% relative reduction in leaf relative water content (RWC; Figure 1g) compared to control plants. In contrast, no significant differences were noted in root length or root DW between salinity-treated and control plants (Figure S1). Taken together, these results indicate that salinity stress, at least for a period of 3–4 weeks, hinders alfalfa cv. Beaver aboveground, but not belowground, growth overall, and leads to a lowering of water content in leaf tissues.

### 2.2. Salinity-Treated Alfalfa Plants Exhibited Reductions in Photosynthesis-Related Parameters Compared to Control-Treated Plants

**Figure 2:**
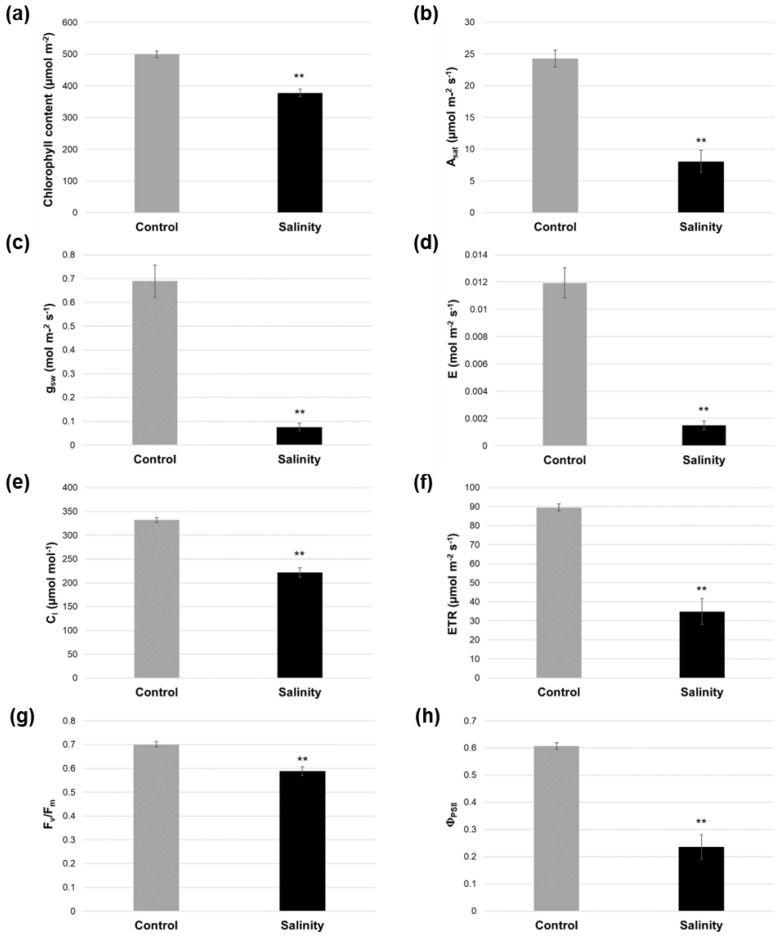
Photosynthesis-related characteristics in alfalfa cv. Beaver under control and saline conditions. (**a**) Chlorophyll content was determined 22 days after initiating treatment (control and salinity, respectively). (**b**–**e**) Light-saturated photosynthetic rate (A_sat_), stomatal conductance (g_sw_), transpiration rate (E), and internal CO_2_ concentration (C_i_) were assessed 22 days after initiating treatment (control and salinity, respectively). (**f**–**h**) Relative electron transfer rate at photosystem II (PSII; ETR), maximum quantum yield of PSII (F_v_/F_m_), and actual quantum yield of PSII (Φ_PSII_) were determined 25 days following the start of treatment (control and salinity). All measurements were made using middle leaflets from first fully expanded trifoliate leaves. For all graphs, blocks represent the mean value of 2 (**b**–**h**) or 3 (**a**) leaves from each of 5 biological replicate plants for each treatment, and bars denote standard errors. Asterisks indicate a statistically significant difference between salinity and control treatments in each graph (**, *p* ≤ 0.01).

In line with the reductions in plant growth observed under salinity stress, significant decreases were also apparent in a variety of photosynthesis-related characteristics approximately 3 weeks following the initiation of treatment (Figure 2). Chlorophyll content, light-saturated photosynthetic rate (A_sat_), stomatal conductance (g_sw_), transpiration rate (E), and internal CO_2_ concentration (C_i_) displayed significant relative reductions of 24.37%, 66.72%, 88.97%, 87.46%, and 33.23%, respectively, in plants grown under saline conditions compared to those grown under control conditions (Figure 2a–e). Furthermore, the maximum quantum efficiency of photosystem II (F_v_/F_m_), which is an indicator of photosynthetic performance and stress, as well as the steady state quantum yield of photosystem II (Φ_PSII_) and relative electron transfer rate at photosystem II (ETR), declined significantly (relative reductions of 15.94%, 61.12%, and 61.12%, respectively) under salinity stress (Figure 2f–h).

### 2.3. Alfalfa Plants under Saline Stress Displayed Altered Osmoprotectant Levels and Antioxidant-Related Parameters Compared to Control Conditions

**Figure 3:**
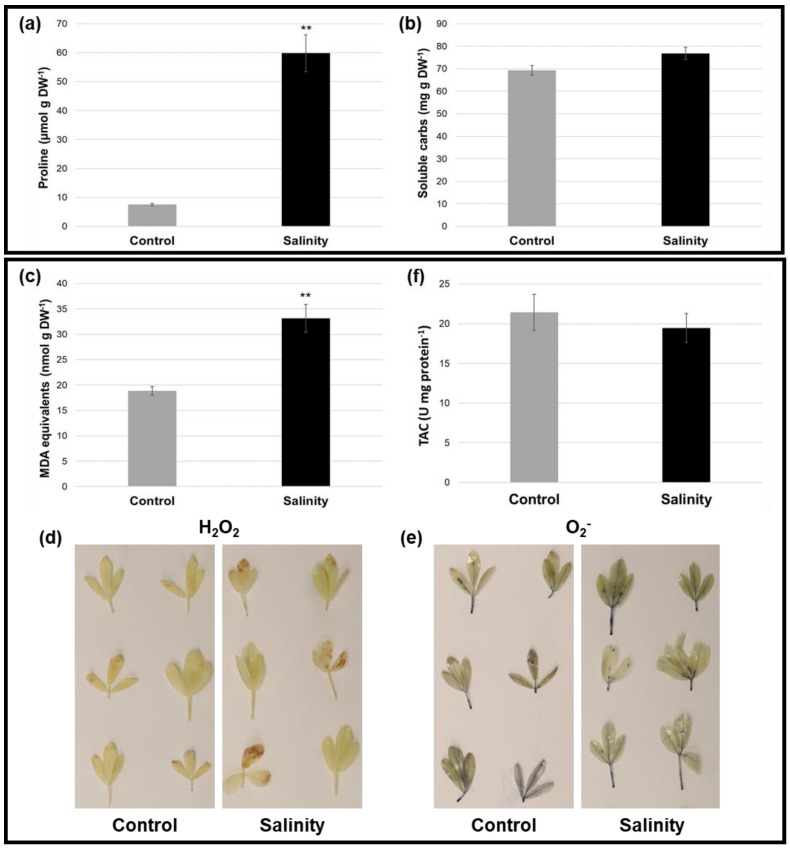
Biochemical and oxidative responses of alfalfa cv. Beaver leaves under control and saline conditions. (**a**–**c**) Proline, soluble carbohydrate, and malondialdehyde (MDA) levels in first fully expanded trifoliate leaves harvested 22 days following the initiation of salinity treatment. (**d**,**e**) Images of representative DAB- and NBT-stained leaves for the estimation of H_2_O_2_ (brown staining) and O_2_^−^ (dark blue staining) levels, respectively, in fully expanded trifoliate leaves (third from the shoot tip) harvested 21 days after the initiation of salinity and control treatments. (**f**) Total antioxidant capacity (TAC) of first fully expanded trifoliate leaves harvested 22 days following the initiation of treatment. For all graphs, blocks represent the mean values of 10 (**a**,**b**), 8 (**c**), or 5 (**f**) biological replicate plants for each treatment, and bars denote standard errors. Proline and MDA assays were carried out in triplicate, while soluble carbohydrate and TAC assays were carried out in duplicate. Asterisks indicate a statistically significant difference between salinity and control treatments in each graph (**, *p* ≤ 0.01).

To gain further insight into the physiological alterations incurred during salinity stress in alfalfa, we also evaluated the levels of osmoprotectants, including proline and soluble carbohydrates, in leaf tissues from plants subjected to control and salinity treatment for approximately 3 weeks. Proline concentration was found to be significantly higher (694.00% relative increase) in alfalfa leaves from salinity-treated plants compared to control-treated plants (Figure 3a). Conversely, although a slight increase in soluble carbohydrate levels was observed under saline compared to control conditions, this difference was not significant (Figure 3b).

In order to assess for possible oxidative damage in alfalfa subjected to salinity stress for approximately 3 weeks, leaves were first evaluated for levels of malondialdehyde (MDA), which is a lipid peroxidation product that is a commonly used marker for oxidative damage to membrane lipids. As expected, alfalfa leaves from saline-treated plants exhibited a significant 75.57% relative increase in MDA content compared to control-treated controls (Figure 3c), which indicates that these plants suffered elevated levels of oxidative damage. In line with this finding, diaminobenzidine (DAB) treatment of leaves yielded more pronounced staining in salinity-treated plants than controls (Figure 3d), which is indicative of higher H_2_O_2_ levels. Conversely, no apparent differences in staining intensity were observed with nitroblue tetrazolium (NBT) treatment (Figure 3e), which suggests that O_2_^−^ levels did not differ dramatically between salinity- and control-treated plants. Similarly, no significant difference in total antioxidant capacity was observed between salinity-treated and control plants (Figure 3f). Taken together, these results suggest that saline-treated alfalfa plants produced elevated levels of H_2_O_2_ but did not increase their total antioxidant capacity under such stress and, as such, exhibited greater oxidative damage.

### 2.4. RNA-Seq Analysis of Leaf Tissue from Salinity- and Control-Treated Alfalfa Plants

To advance our understanding of alfalfa’s molecular response to salinity stress, we carried out RNA-Seq on leaves from plants grown under each treatment for 25 days. Between 126,016,842 and 231,379,036 raw reads were obtained per sample, and a mean of 72.35% of reads were mapped to the *M. truncatula* reference genome (v4.0; Table S1). An average of 19,184 and 19,216 mapped expressed transcripts were identified in control- and salinity-treated samples, respectively (Table S1). Principal component analysis (PCA) analysis of trimmed mean of M-values (TMM)-normalized reads led to the identification of two distinct groups, confirming the similarity of the biological replicates within the control and salinity groups, respectively (Figure S2a). The analysis also indicates that the differential expression signal is not driven by the batch factor. Between salinity and control conditions, we identified 1233 significant differentially expressed genes (DEGs), including 680 that were down-regulated under salinity treatment compared to control treatment, and 553 that were up-regulated (File S1; Figure S2b). A comparison of log_2_ fold-changes derived from qRT-PCR and RNA-Seq for eight selected transcripts confirmed the validity of the RNA-Seq results, with a correlation coefficient of 0.98 across methods (Figure S3).

**Figure 4:**
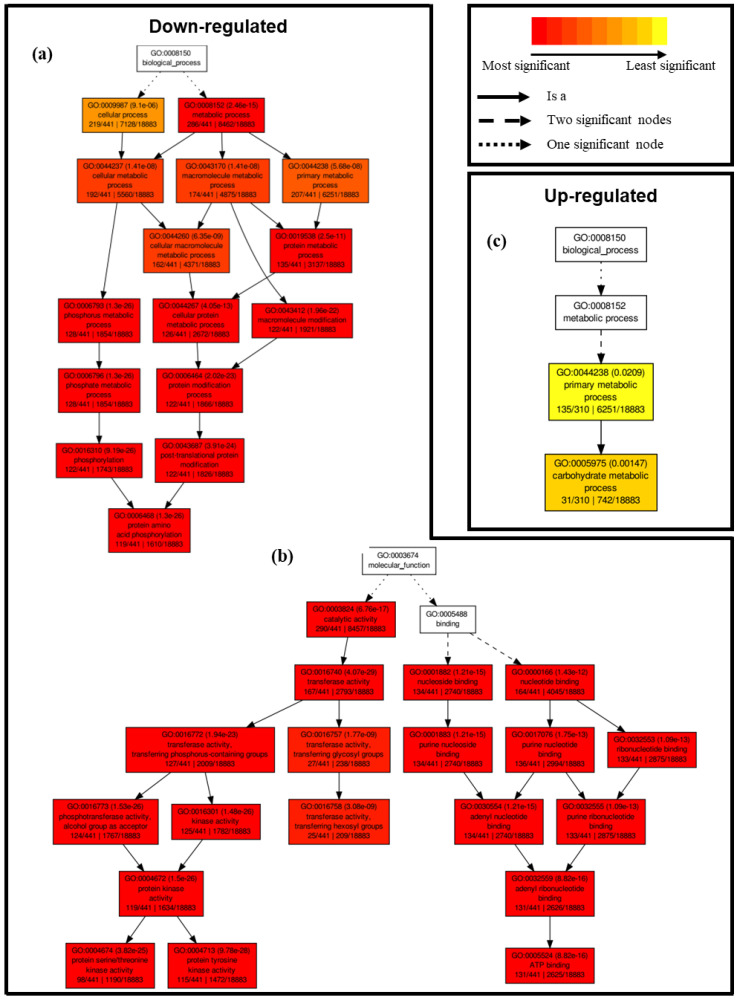
Singular enrichment GO term analysis of transcriptomic changes incurred under salinity compared to control conditions in alfalfa cv. Beaver leaves. (**a**) Biological process GO term category for down-regulated genes. (**b**) Molecular function GO term category for down-regulated genes. (**c**) Biological process GO term category for up-regulated genes. Box color indicates the significance of changes relating to each GO term. Analyses were conducted using a significance level of 0.05.

For genes that were down-regulated under salinity conditions compared to control treatment, singular enrichment analysis led to the identification of 34 significantly enriched gene ontology (GO) terms (Figure 4a,b). In the biological process category, this included GO terms such as ‘protein metabolic process’, ‘phosphorylation’, and ‘post-translational protein modification’, for example (Figure 4a). In the molecular function category, GO terms such as ‘kinase activity’, ‘nucleotide binding’, and ‘transferase activity’ were identified (Figure 4b). No significantly enriched GO terms were observed in the cellular components category for down-regulated DEGs. Conversely, only two significantly enriched GO terms were distinguished in the case of up-regulated DEGs, including ‘primary metabolic process’ and ‘carbohydrate metabolic process’ in the biological process category (Figure 4c).

#### 2.4.1. Differential Transcriptional Responses in General Metabolic Pathways between Saline and Control Conditions

**Figure 5:**
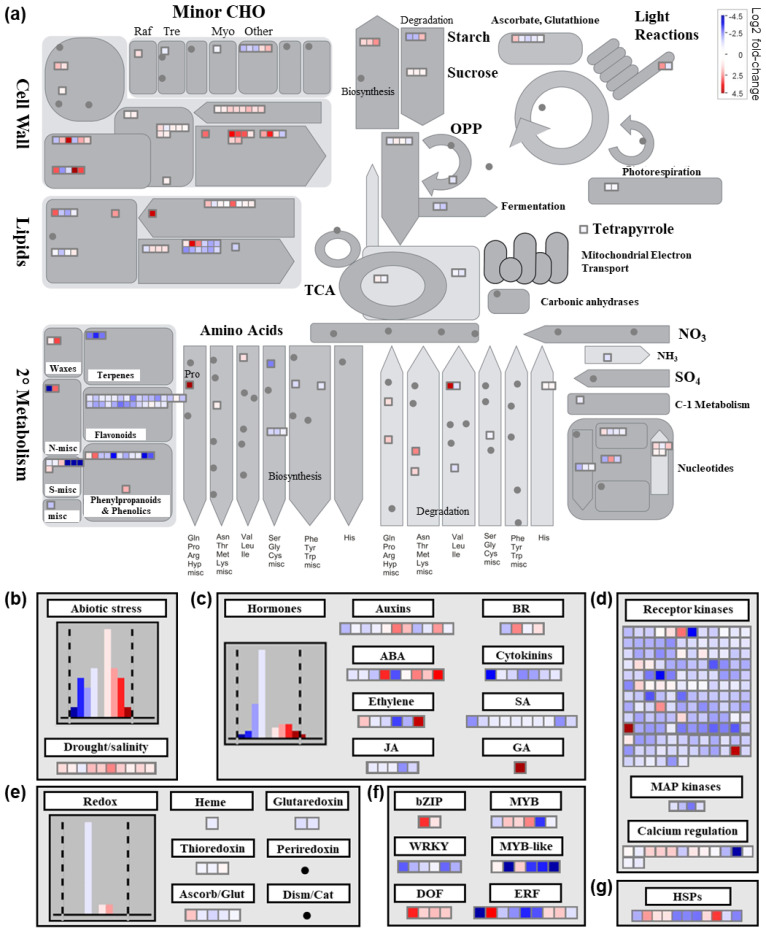
Transcriptional differences among various metabolic pathways and gene families under salinity compared to control conditions in alfalfa cv. Beaver leaves. (**a**) General metabolic pathways; (**b**) abiotic stress-related pathways; (**c**) hormone metabolism-related pathways; (**d**) signaling-related pathways; (**e**) redox-related pathways; (**f**) stress-related transcription factor families; and (**g**) heat shock proteins. Small blue boxes denote down-regulated DEGs, while red/pink boxes indicate up-regulated genes. Gray boxes/shapes represent different pathway groupings according to the MapMan program, and small circles indicate that no DEGs were observed in a particular pathway. ABA, abscisic acid; Ascorb/Glut, ascorbate/glutathione; BR, brassinosteroids; CHO, carbohydrates; Dism/Cat, dismutase/catalase; ERF, ethylene response factors; GA, gibberellic acid; HSPs, heat shock proteins; JA, jasmonic acid; misc, miscellaneous; OPP, oxidative pentose phosphate pathway; Pro, proline; SA, salicylic acid; TCA, tricarboxylic acid cycle.

To gain further insight into the transcriptional response of alfalfa under salinity stress, we also carried out MapMan pathway analysis of DEGs between treatments. In terms of general metabolic pathways (Figure 5a), there was an abundance of genes that were up-regulated in cell wall-related pathways, including genes encoding various cellulose synthase and cellulose synthase-like isomers (e.g., Medtr3g030040, Medtr3g107520, Medtr4g077910, and Medtr8g092590), as well as Medtr7g055600, which has a putative role in pectin biosynthesis (Figure 5a; File S2). Several genes involved in minor carbohydrate metabolic pathways were also differentially expressed under salinity treatment, including the down-regulation of a gene encoding a protein with a putative function in trehalose biosynthesis (Medtr0034s0170; File S2).

Numerous genes involved in lipid metabolism were also differentially expressed under salinity stress in alfalfa (Figure 5a). For example, a gene encoding a choline kinase (Medtr6g040230) that is involved in phospholipid biosynthesis was found to be down-regulated under salinity stress in alfalfa in the current study (File S2). Conversely, Medtr1g111880, which encodes an omega-6 fatty acid desaturase, was highly up-regulated (4.12 log_2_ fold change) under salinity stress. Numerous genes involved in lipid degradation were also differentially expressed following salinity treatment, including the up-regulation of Medtr5g023050, which encodes a protein with phospholipase D activity (File S2). Furthermore, two genes encoding enzymes known to be involved in wax biosynthesis were also differentially regulated (both up-regulated) under salt treatment in alfalfa, namely, 3-ketoacyl-CoA synthase 4 (KCS4; Medtr4g070270) and ECERIFERUM 1 (CER1; Medtr8g009560) (File S2). Similarly, Medtr1g086600, which encodes long-chain acyl-CoA synthase 1 (LACS1) and is involved in the production of cuticular wax and cutin, was also up-regulated under salinity treatment (File S1).

Various genes involved in amino acid metabolism were also differentially expressed under saline stress, including a gene encoding a delta 1-pyrroline-5-carboxylate synthetase (P5C5; Medtr4g020110), which is a rate-limiting enzyme in proline biosynthesis and was highly up-regulated (4.93 log_2_ fold-change; File S2).

#### 2.4.2. Differential Transcriptional Responses in Abiotic Stress- and Redox-Related Pathways between Saline and Control Conditions

The majority of DEGs falling within the abiotic stress pathway bin were up-regulated in salinity-treated plants compared to controls, with all but one DEG in the drought/salt response pathway bin being up-regulated (Figure 5b). Conversely, Medtr6g092970, which encodes HIGH-AFFINITY POTASSIUM TRANSPORTER 1 (HKT1), was significantly down-regulated under salinity stress (File S1). Similarly, genes encoding two inwardly rectifying potassium channel subunits (Medtr2g019530 and Medtr8g446430) and a potassium efflux antiporter (Medtr8g031550) were also down-regulated under salinity stress in alfalfa leaves, while a gene encoding a potassium outwardly rectifying channel protein (Medtr5g077770) was up-regulated (File S1). Genes encoding ABC transporters, on the other hand, were more often up-regulated under salinity stress (Medtr4g057795, Medtr8g015980, Medtr0019s0020, Medtr5g033320, Medtr1008s0010, Medtr0196s0020, Medtr8g093840, Medtr1g025560, Medtr1g050525, Medtr8g059150, Medtr4g124050) than down-regulated (Medtr3g011840, Medtr5g029750, Medtr4g109720, Medtr8g107410, Medtr3g011820, Medtr3g092500, Medtr3g040090) (File S1).

DEGs encoding proteins involved in cell signaling pathways, such as receptor kinases, MAP kinases, and calcium regulation, were largely down-regulated in alfalfa leaves under salinity stress conditions (Figure 5d). For example, this included ten genes that encoded FERONIA-like proteins, with nine that were down-regulated (Medtr7g015230, Medtr7g015240, Medtr7g015250, Medtr7g015280, Medtr7g015290, Medtr7g015310, Medtr7g015320, Medtr7g015390, and Medtr7g015620) and only one that was up-regulated (Medtr7g073660; File S2). In addition, a gene encoding a MAP kinase kinase 2 (MKK2) homolog (Medtr2g040510) and calcineurin B-like protein (Medtr8g027620) were down-regulated in alfalfa leaves under salinity treatment (File S2).

Similarly, DEGs involved in redox-related pathways were also mainly down-regulated under salinity conditions (Figure 5e; File S2), and no DEGs were observed in periredoxin or dismutase/catalase metabolism pathway bins in this study (Figure 5e). The vast majority of DEGs involved in terpene, flavonoid, and phenylpropanoid/phenolic metabolic pathways were also down-regulated in alfalfa under salinity stress in the current study (Figure 5a, File S2). In addition, eleven genes encoding heat shock proteins were fairly equally divided in terms of being up- and down-regulated under salinity stress in alfalfa leaves (Figure 5g), while a single gene encoding a late embryogenesis abundant (LEA; Medtr6g081930) protein was up-regulated (File S1).

#### 2.4.3. Differential Transcriptional Responses in Phytohormone Metabolism-Related Pathways and Genes Encoding Transcription Factors between Saline and Control Conditions

With respect to phytohormone metabolism overall, the majority of DEGs were down-regulated under saline conditions compared to control treatment (Figure 5c). Indeed, every DEG falling within jasmonic acid, cytokinin, and salicylic acid metabolic pathways was significantly down-regulated under salinity stress (Figure 5c; File S2). Furthermore, various genes encoding proteins/enzymes involved in auxin, abscisic acid (ABA), brassinosteroid, ethylene, and gibberellic acid metabolism/response were also differentially regulated under saline stress (Figure 5c, File S2).

DEGs encoding transcription factors were found to be both up- and down-regulated to a fairly equal extent under salinity treatment (Figure 5f). In terms of transcription factor families with known functions in abiotic stress response, all DEGs encoding bZIP and DOF transcription factors were up-regulated, while all DEGs encoding WRKY transcription factors were down-regulated under salt stress (Figure 5f; File S2). Conversely, DEGs falling within MYB, MYB-like, and ERF families were both up- and down-regulated (Figure 5f, File S2).

### 2.5. Metabolomic Response in Leaf Tissues from Salinity- and Control-Treated Alfalfa Plants

**Figure 6:**
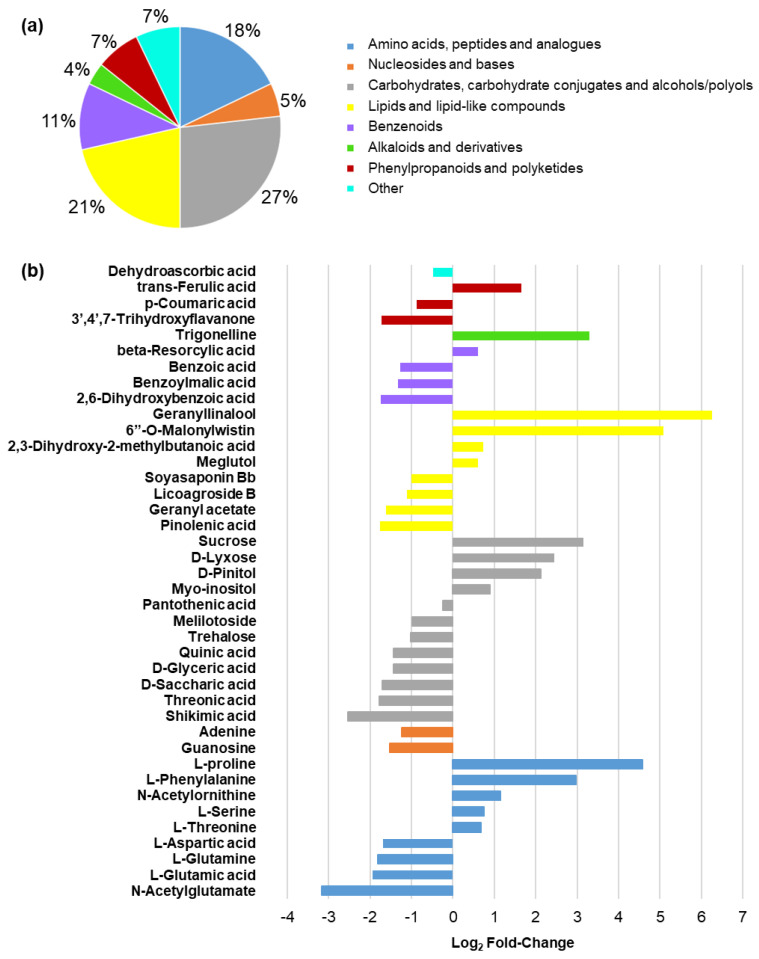
Variation in leaf metabolite levels under salinity compared to control conditions in alfalfa cv. Beaver. (**a**) Proportion of differential metabolites of various classifications across conditions. (**b**) Examples of various metabolites with levels that differ significantly between control and saline conditions. Block color reflects metabolite classification as indicated in (**a**).

We detected 18,026 and 10,627 metabolic features in the reversed phase (+) (RP(+)) and HILIC(−) analyses, respectively. Following filtering, this corresponded to a total of 4472 and 2674 high-quality metabolic features. These high-quality features were then used for PCA, where a clear separation of the two groups of samples was observed for HILIC(−) data, as well as the similarity of global profiling of non-polar metabolites in RP(+) (Figure S4a). Our results suggest that polar metabolites may be more relevant in the response to salinity treatment than non-polar metabolites. Initial analyses indicated that numerous metabolites were differentially abundant between conditions (Figure S4b). Following de-replication and manual inspection, we identified 60 significant annotated metabolites in total (File S3). Of these, 56 could be classified into various groups, including amino acids, nucleosides and bases, carbohydrates and carbohydrate-conjugates, alcohols and polyols, lipids and lipid-like compounds, benzenoids, phenylpropanoids, and alkaloids and derivatives (Figure 6a). As a group, carbohydrates, carbohydrate conjugates, and alcohols/polyols made up the largest proportion of differential metabolites between growth conditions, with lipids and lipid-like compounds, and then amino acids, peptides, and analogues making up the next highest proportions (Figure 6a).

In the case of carbohydrates and polyols, sucrose, D-lyxose (also known as pectin), myo-inositol, and pinitol, for example, all increased significantly under salinity stress, while trehalose and pantothenic acid levels significantly decreased (File S3). In addition, both quinic acid and shikimic acid, which function within the shikimate pathway, were significantly down-regulated under salinity stress (File S3), while metabolites generated downstream of this pathway were either up-regulated (e.g., phenylalanine and ferulic acid) or down-regulated (*p*-coumarate, 3′,4′,7-trihydroxyflavanone, and benzoic acid) (File S3). In terms of lipids and lipid-like compounds, levels of the polyunsaturated pinolenic acid were significantly elevated following salt treatment (File S3), while geranyl acetate and soyasaponin Bb levels significantly declined under this stress (File S3), for instance. With respect to amino acids other than phenylalanine, L-glutamine, L-aspartic acid, and L-glutamic acid all decreased significantly under salt stress, while L-proline, L-serine, L-threonine, and N-acetylornithine significantly increased, for example (File S3). Various other compounds of interest also differentially accumulated following salt treatment, including guanosine, adenine, and dehydroascorbic acid, which decreased significantly, as well as trigonelline, which increased significantly (File S3).

### 2.6. Joint Pathway Enrichment Analysis

**Figure 7:**
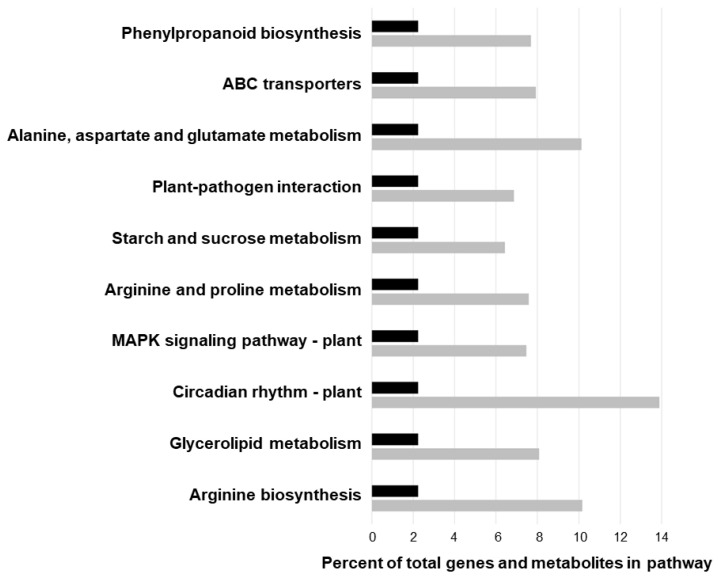
Joint pathway analysis of differential expression and metabolite levels in alfalfa cv. Beaver leaves under salinity compared to control conditions. All pathways listed had FDRs under 0.05. Blocks indicate the percentage of genes and metabolites that were affected by salinity treatment in each pathway, with black blocks denoting the percentages that would be expected if there were no enrichment, and gray blocks indicating actual percentages.

To further understand the metabolic pathway changes incurred in alfalfa under salinity stress, we performed joint-pathway analysis of significantly regulated metabolites and DEGs from our RNA-Seq experiment (Figure 7; File S4). In terms of metabolic pathways, phenylpropanoid biosynthesis, alanine, aspartate, and glutamate metabolism, starch and sucrose metabolism, arginine and proline metabolism, glycerolipid metabolism, and arginine biosynthesis were significantly affected with false discovery rates (FDRs) below 0.05. Furthermore, several other pathways in salinity-treated alfalfa plants were also found to be significantly enriched, including ABC transporters, plant–pathogen interaction, the mitogen-activated protein kinase (MAPK) signaling pathway, and circadian rhythm (Figure 7; File S4).

## 3. Discussion

Soil salinization is increasing globally and is particularly problematic for crops with high water demands, such as alfalfa, which require irrigation in many growing regions [37]. Although alfalfa is deemed to be moderately resilient to salinity [38], it can suffer considerable biomass yield penalties when subjected to salt stress [39,40]. Like most plant species, these detrimental effects stem from physiological disturbances that occur as a result of osmotic and secondary oxidative stress, as well as ion toxicity and a diminished capacity for nutrient uptake [1]. While a general framework for salinity stress response in plants has been unraveled over recent years, including various signal transduction pathways, ion transport/compartmentalization mechanisms, and processes to counter osmotic and oxidative damage [41,42], gaps remain in our understanding of this process, particularly in non-model species such as alfalfa. As such, we carried out in-depth comparative physiological evaluations of alfalfa cv. Beaver, which exhibits limited salt tolerance [43,44], as well as transcriptomic and metabolomic assessments of leaf tissues from mature plants, following long-term salt (3–4 weeks of salinity treatment) and non-saline control treatment as a means of advancing our understanding of salt response mechanisms in this species.

As expected, alfalfa plants in our study were significantly shorter, with a smaller number of shoots, shorter internodes, and lower aboveground FW and DW following salinity treatment (Figure 1a–f), which corresponds with previous findings in this species [13,45,46]. Conversely, we did not observe any significant differences in belowground characteristics such as root length or DW under salinity stress (Figure S1). While the growth of alfalfa roots has previously been found to be negatively affected under salinity conditions, this has not always been the case [45], and root growth tends to be less severely impacted than shoot growth overall [13,47,48], which highlights the importance of understanding aboveground responses to salinity.

The decline in aboveground growth corresponded with significant reductions in a variety of photosynthetic-related characteristics, including chlorophyll content, light-saturated photosynthetic rate, stomatal conductance, transpiration rate, internal CO_2_ concentration, maximum quantum yield of photosystem II, actual quantum yield of photosystem II, and the relative electron transfer rate at photosystem II (Figure 2a–h). Decreases in photosynthesis are typically observed following an extended period of salinity in plants [49,50], and this tends to correlate with the transcriptional modulation of various photosynthesis-related genes in alfalfa, at least under short-term salt stress [24,51]. However, very few genes with functions in any component of the photosynthetic process were differentially expressed in alfalfa leaves following longer-term salinity treatment in the current study (Figure 5a). This suggests that decreases in photosynthetic parameters were caused, at least in part, by stress-related impairment of the photosynthetic apparatus itself, as well as potential stomatal closure and a concomitant decrease in the uptake/fixation of CO_2_, which are typical salinity-derived effects in plants [52].

Exposure to root zone salinity triggers a series of interconnected signaling and transduction events in plants, which are often initiated by an increase in cytosolic Ca^2+^ [53]. Interestingly, it has been shown previously that signal perception- and transduction-related genes tend to be up-regulated very early on during salinity stress in alfalfa root tips [26]; however, following approximately 20 months of treatment with mixed salt irrigation water, very few changes were observed in the expression of Ca^2+^ signaling-related genes in the leaves of either salt-tolerant or -sensitive genotypes [23]. Following salinity treatment in the current study, a relatively large number of genes involved in cell signaling and transduction pathways were differentially expressed under salinity stress in alfalfa leaves, with the vast majority being down-regulated (Figure 5d). These discrepancies among studies suggest that considerable differences may exist in the transcriptional regulation of signaling and transduction pathways in alfalfa in response to salinity stress depending on genotype, tissue, and/or length of exposure.

Similarly, the MAPK signaling pathway, which is an interconnected signal transduction pathway that plays a role in salinity response in plants [54], was significantly enriched in our integrated pathway analysis (Figure 7), and all DEGs within this pathway were down-regulated (Figure 5d), including a *MKK2* homolog (File S2). In addition, nine genes encoding FERONIA-like proteins, which are receptor kinases that mediate Ca^2+^ transients and are involved in the sensing of salt-induced damage to cell walls, as well as the maintenance of their integrity [55], were also down-regulated in response to salinity in alfalfa leaves in the present study (File S2). Given that the over-expression of both *FERONIA* (*FER*) and *MKK2* homologs has been shown to enhance salinity tolerance in plants previously [56,57,58], and both Arabidopsis *fer* and *mkk2* mutants exhibit hypersensitivity to salt [55,57], it is feasible that maintaining or enhancing their expression in alfalfa under salinity stress could provide a potential target for improving tolerance in this species.

Our transcriptomic data also suggests that cell wall remodeling was likely taking place in alfalfa following salinity treatment, since we observed substantial modulations in the expression of cell wall metabolism-related genes, with the majority being up-regulated (Figure 5a; File S2). This included multiple cellulose synthase-encoding genes, as well as a gene encoding a putative member of the QUASIMODO family, members of which have been suggested to be involved in pectin biosynthesis previously (File S2) [59]. This latter finding corresponded with a significant increase in D-lyxose (pectin) in alfalfa leaves under salinity (Figure 6b; File S3). Although there is some evidence that both cellulose and pectin levels are positively associated with abiotic stress tolerance [60,61], substantial gaps remain in our understanding of particular cell wall polysaccharides in the context of salinity response, and further investigation is warranted in this area.

When a plant is exposed to NaCl in the soil, salt ions move into the root and are then transported to shoots, where they eventually accumulate in leaves and disrupt K^+^/Na^+^ balance, which leads to ion toxicity and nutrient deficiencies [17,62]. Plants possess mechanisms to moderate ionic stress and toxicity, and maintaining cellular ion homeostasis (particularly the K^+^/Na^+^ ratio) is an important characteristic of plants exhibiting high levels of salt tolerance [63]. Within shoots, such mechanisms mainly comprise the extrusion of Na^+^ from the cytoplasm into the apoplast, the potential export of Na^+^ from shoots back into roots via the phloem, and the sequestration of ions into vacuoles, which typically rely on a variety of ion transporters [64]. The SOS pathway is known for its involvement in the efflux of Na^+^ out of plant cells under salinity stress [65]. This pathway first involves the binding of Ca^2+^ by SOS3 (also known as calcineurin B-like 4 [CBL4]) in the plasma membrane, which subsequently binds and activates the serine/threonine protein kinase SOS2 (also known as CBL interacting protein kinase 24 [CIPK24]). SOS2 can then phosphorylate the Na^+^/H^+^ antiporter SOS1 (also known as sodium/hydrogen antiporter 7 [NHX7]), which extrudes Na^+^ from the cell in exchange for H^+^ [63]. In line with this, salt tolerance in a variety of plant species has been linked with higher expression levels of *SOS1*, *SOS2,* and *SOS3,* and an associated elevated capacity to exclude Na^+^ from shoots [66,67]. Interestingly, it has been shown previously that the SOS1-dependent pathway might be more important in alfalfa roots than shoots [24,46], which corresponds with our transcriptomic data suggesting that none of the three *SOS* gene orthologs (putatively Medtr2g038400, Medtr4g114670, and Medtr3g091440) were differentially expressed in alfalfa leaves under salt conditions. However, a gene encoding a calcineurin B-like protein (Medtr8g027620), which is related to Arabidopsis *SOS3*, was down-regulated (File S1).

Genes encoding vacuolar membrane NHXs functioning in the compartmentalization of cytosolic Na^+^ into vacuoles [64] have been shown to be induced in alfalfa leaves in response to short-term salinity stress (a single treatment of 500 mM NaCl followed by harvesting 7 days later) [24]. However, we did not observe any differential regulation of genes encoding vacuolar NHX homologs in the present study, which could be due to genotype-specific effects, treatment conditions, length of exposure, or plant growth stage. Conversely, we did observe the down-regulation of *HKT1* (File S1), which functions in the maintenance of Na^+^ and K^+^ homeostasis in plants via the retrieval/diversion of Na^+^ from the xylem and/or the loading of excess Na^+^ into the phloem as a means of protecting shoots from Na^+^ toxicity [68,69]. Similarly, two genes encoding inwardly rectifying K^+^ channel subunits, which are known to specifically facilitate K^+^ uptake and transport in plant cells, as well as one encoding a K^+^ efflux antiporter, which can function in promoting long-distance K^+^ transport from roots to shoots [70], were also down-regulated in alfalfa leaves in the current study (File S1). Intriguingly, these channels/antiporters can also function in the movement of K^+^ into and out of guard cells to moderate stomatal aperture, which is also relevant in the context of leaf salinity response [70]. In addition to channels and transporters that are specific to particular ions, ABC transporters are also known to play a role in salinity response in plants and comprise a large superfamily that is involved in the energy-dependent transport of many different substances across membranes [71]. As has been found previously in other alfalfa cultivars under short-term salinity [51], genes encoding ABC transporters were most often up-regulated in alfalfa cv. Beaver leaves under salinity stress (File S1), and joint pathway analysis indicated a corresponding significant enrichment in ABC transporters (Figure 7).

The generation of ROS, such as H_2_O_2_ and O_2_^-^, also tends to be induced in plants following exposure to salinity stress, particularly in salt-sensitive genotypes [26,72,73]. While ROS are typical by-products of metabolic pathways such as respiration and photosynthesis and also function as key secondary signaling molecules [74], excessive levels that can result from stress conditions lead to cellular damage via the oxidation of proteins, nucleic acids, and lipids [75]. Following salinity treatment in the present study, H_2_O_2_, but not O_2_^−^, levels appeared to increase in alfalfa leaves (Figure 3d,e). This increase in H_2_O_2_ corresponded with a significant augmentation in the levels of MDA (Figure 3c), which indicates higher levels of lipid peroxidation, following approximately 3 weeks of salinity treatment.

To lessen the deleterious effects of excessive ROS produced under stress, plants typically activate the production of various ROS-scavenging/detoxifying antioxidants, including those that are enzymatic and non-enzymatic in nature [74]. Following salinity stress, alfalfa characteristically exhibits increased activities of various enzymatic antioxidants such as superoxide dismutase, peroxidase, and ascorbate peroxidase [25,26,76]. However, this is not always the case [77], and it is possible that these effects are genotype-specific [24,25,76], which could explain the overall lack of modulation of total antioxidant capacity in salinity-treated leaves in the present study (Figure 3f). Correspondingly, we also only observed the differential transcriptional regulation of a very small number of genes encoding proteins with enzymatic antioxidant-related functions following salinity stress (Figure 5e), and in cases where genes were differentially expressed, they were mainly down-regulated. Similarly, the vast majority of DEGs involved in the production of various other secondary compounds with putative antioxidant properties, such as terpenes/terpenoids and polyphenolics [78,79,80], were also down-regulated following salinity stress (Figure 5a), which correlated with declines in the levels of the monoterpene geranyl acetate and triterpenoid soyasaponin, dehydroascorbic acid, and multiple metabolites in the shikimate pathway (quinic acid and shikimic acid), as well as the downstream ρ-coumarate and flavonoid 3′,4′,7-trihydroxyflavanone, in alfalfa leaves following salinity stress (Figure 6b, File S3). Given the apparent overall reduction in antioxidant-related gene expression and metabolite levels, it is unclear why no decrease in antioxidant capacity was observed in this study. However, since little correlation appears to exist between transcriptional levels and enzymatic antioxidant activities in plants [81,82,83], possibly due to as of yet unknown post-translational regulatory mechanisms [84,85], it is possible that increased enzymatic activities could be offsetting reductions in non-enzymatic antioxidants under long-term salinity stress in alfalfa. In line with this, it has been found previously that the treatment of plants with ferulic acid, the levels of which were significantly increased under salinity treatment in the current study, enhances the activities of various antioxidant enzymes [86,87]. Although further study will be required to elucidate precise antioxidant effects in alfalfa grown under saline conditions, a similar phenomenon could feasibly be occurring in this case.

Plants also typically trigger the increased production of phytohormones such as ABA, which functions as a key signal transduction component in abiotic stress response to link environmental prompts and development under salinity [88]. While ABA levels tend to increase following salinity stress in plants [89], this effect can wane over time during prolonged exposure in salt-sensitive plants [90], which corresponded with the fact that we did not observe any significant alteration in ABA levels in alfalfa leaves following salinity treatment (File S3) and ABA-related DEGs were both up- and down-regulated (Figure 5c). In addition to ABA-related pathways, various genes related to the metabolism of other phytohormones were also differentially expressed in alfalfa leaves under salinity in this study (Figure 5c). Interestingly, all DEGs falling within jasmonic acid, cytokinin, and salicylic acid metabolic pathways were down-regulated under salinity (Figure 5c). However, although benzoic acid, which is a precursor for salicylic acid biosynthesis [91], was present at reduced levels under salinity stress (Figure 6b; File S3), no significant differences in the levels of salicylic acid, jasmonic acid, or cytokinin were observed in our metabolomic assessment (File S3), which suggests that they may not provide a major function in the later stages of salinity response in alfalfa cv. Beaver.

Saline conditions in the region of the root zone also have an immediate effect on plants due to a decrease in their capacity for water uptake, which leads to osmotic stress [38]. In the present study, we noted significant reductions in relative leaf water contents in salinity-treated compared to control-treated alfalfa (Figure 1g), which is typical of plants grown in a saline environment [92,93]. As a means of countering reduced water availability under salinity stress, plants characteristically accumulate compatible solutes (also known as osmoprotectants) such as various sugars, polyols, and amino acids, which also often provide a dual role as free radical scavengers [94]. For example, total soluble carbohydrates tend to increase under salt stress in plants, including alfalfa [95]. However, while we observed a slight increase in the overall levels of soluble carbohydrates in alfalfa leaves following approximately 3 weeks of salinity stress, this difference was not significant (Figure 3b). Since salinity-induced changes in soluble carbohydrates tend to be genotype-specific [96,97], and in certain genotypes initial increases are not maintained after a couple of weeks [97], this could also be the case in alfalfa cv. Beaver. However, when carbohydrates were observed on an individual basis, we noted both increases and decreases under salinity. For instance, while elevated levels of sucrose were present in the leaves of salinity-treated plants compared to controls in the current study, levels of trehalose were significantly reduced (Figure 6b, File S3), and this corresponded with the down-regulation of a gene encoding trehalose-6-phosphate synthase (Files S1 and S2), which catalyzes the first step of trehalose biosynthesis in plants [98]. Given the importance of trehalose in terms of conferring resilience to saline conditions [99,100], these findings suggest that trehalose may provide an interesting target of study for further improving salinity tolerance in alfalfa.

The accumulation of cyclic polyols, such as myo-inositol and its derivative pinitol, in leaves is also known to be characteristic of salinity response in many plants [101], which was also observed in the present study (Figure 6b, File S3). As is the case with trehalose, enhanced levels have been shown to provoke improvements in salinity tolerance in a variety of plant species previously, at least in part through their roles as osmoprotectants and antioxidants [102,103,104]. Similarly, levels of the alkaloid osmoprotectant, trigonelline, which is known to accumulate during osmotic stress in certain plant species [105,106] and has been suggested to play a role in abiotic stress tolerance previously [105], were also elevated under salinity in alfalfa leaves in the current study (Figure 6b; File S3). Taken together, these results suggest that the accumulation of these metabolites may be a common mechanism used across alfalfa genotypes to withstand salt stress [107,108,109].

Following approximately 3 weeks of salinity stress, levels of the well-known osmoprotectant amino acid proline, as well as the expression of the rate-limiting gene in its biosynthesis (P5C5) [110], increased substantially in alfalfa leaves (Figure 3a and Figure 6b; Files S1 and S3), which is a characteristic response in plants including alfalfa [111,112]. As with other osmoprotectants, previous reports have demonstrated that the transgenic enhancement of proline levels in plants leads to improved salinity tolerance [113,114,115], which highlights their importance in the context of this trait and suggests that the accumulation of osmoprotectants in alfalfa leaves under salinity stress might contribute to the moderate levels of tolerance observed in this species. The metabolism of various other amino acids has also been suggested to play a role in plant cells during plant stress response; however, their precise roles remain to be elucidated [116,117]. In the current study, we observed significant increases in the levels of N-acetylornithine, threonine, serine, and phenylalanine, but significant decreases in aspartic acid, glutamate, N-acetylglutamate, and glutamine (Figure 6b). While it is possible that decreases in some of these amino acids may be related to increases in proline content, further research will be required to fully unravel their specific functions during salt stress.

In addition to modulating the levels of osmoprotectants, plants also attempt to maintain osmotic potential in their leaves during salinity stress by minimizing water loss through stomatal closure, as well as modifying cuticle and epicuticular wax composition and/or contents to reduce residual transpiration [118]. In this study, we observed the up-regulation of several genes with potential functions in cutin and wax production, including *KCS4*, *CER1*, and *LACS1* [119,120,121] (Figure 5a, Files S1 and S2), which could feasibly contribute to the moderate salinity tolerance of alfalfa. In addition, alterations in the expression of genes involved in other lipid-related pathways, as well as the levels of various lipid metabolites, were also evident in alfalfa leaves following salinity stress (Figure 5a and Figure 6b, File S3). Such changes could potentially be involved in the remodeling of membrane lipids under salinity, which has previously been found to occur in plant species, including alfalfa [36,122]. In the current study, a gene encoding a choline kinase, which is involved in phosphatidylcholine biosynthesis and has been found previously to be up-regulated under high salt conditions in Arabidopsis [123], was down-regulated under salinity stress in alfalfa leaves (File S2), which could lead to an associated reduction in membrane integrity. Conversely, a gene encoding an omega-6 fatty acid desaturase, which is known to be involved in the synthesis of 18:2 fatty acids [124], was highly up-regulated under salinity stress (File S1). This latter finding correlates well with previous studies in which the expression of the omega-6 fatty acid desaturase *FAD2* was up-regulated under salinity stress in alfalfa leaves [36], and the suggestion that fatty acid desaturation by FADs may provide an important adaptive mechanism to deal with salt stress in alfalfa through the effect of polyunsaturated fatty acid levels on membrane fluidity [125]. Numerous genes involved in lipid degradation were also differentially expressed following salinity treatment, including the up-regulation of a gene encoding a protein with phospholipase D activity (File S2). Interestingly, phospholipase D proteins, which regulate the production of the important signaling lipid phosphatidic acid, have also been shown to function in salinity stress response in plants [126] and tend to be up-regulated under this type of stress [36]. However, their precise role in salinity stress response and/or tolerance remains to be determined.

Salt stress is also known to increase the production of proteins with protective roles, including LEAs and heat shock proteins, for example [127], and elevated levels of expression have been linked to salinity tolerance in plants [128,129,130,131,132]. In the present study, we observed the substantial up-regulation of a single gene encoding a LEA protein (File S1), as well as both the up- and down-regulation of various genes encoding heat shock proteins (Figure 5g; Files S1 and S2). Furthermore, various transcription factors also play crucial roles in salinity response in plants, including alfalfa, due to their responsiveness to signaling cascades and the downstream transcriptional regulation of numerous stress-related genes [22,24]. Intriguingly, we found all differentially expressed *bZIP* and *DOF* genes to be up-regulated in alfalfa leaves under saline treatment in the present study, while all *WRKY* genes were down-regulated (Figure 5f). Conversely, genes encoding *MYB*, *MYB*-like, and *ERF* transcription factors were both up- and down-regulated (Figure 5f). While it is well known that the over-expression of genes encoding several protective proteins and transcription factors from alfalfa, including *MsLEA3-1* [133], *MsHSP23* [134], *MsZIP* [135], *MsMYB4* [22], *MsMYB2L* [136], *MsERF11* [137], *MsERF8* [138], and *MsWRKY11* [139], has led to enhancements in salinity tolerance in plants previously, the specific functions of the genes identified here in terms of salinity response have yet to be unraveled.

In conclusion, our findings suggest that during the later stages of salinity response following several weeks of exposure to the stress, various molecular and metabolic adaptations have taken place in photosynthetic tissues as a means of protecting the cells from, and almost certainly also as a result of, osmotic, oxidative, and ionic stress. These include potential changes to cell membranes and walls, cuticular and/or epicuticular waxes, osmoprotectant levels, antioxidant-related metabolic pathways, and the expression of genes encoding ion transporters, protective proteins, and transcription factors. While several of these alterations have likely contributed to alfalfa’s moderate capacity to tolerate saline conditions, a number of mechanisms may be detrimental and could provide ideal targets for manipulation in order to better understand salinity response/tolerance mechanisms in this species, and potentially for the further improvement of this trait downstream.

## 4. Materials and Methods

### 4.1. Plant Growth Conditions

Seeds of *M. sativa* cv. Beaver (provided by Dr. Surya Acharya, Agriculture and Agri-Food Canada, Lethbridge Research and Development Centre), which exhibits limited salt tolerance [43,44], were grown individually in Cornell soilless potting mix [140] in square pots (10.5 cm across and 12.5 cm in height) under greenhouse conditions with supplemental light providing a 16 h/8 h photoperiod, day/night temperatures of approximately 25/15 °C, and a light intensity of approximately 425 µmol.m^−2^.s^−1^. Due to alfalfa’s outcrossing nature, all experiments were carried out using biological replicates of a single genotype derived from vegetative stem cuttings. Plants were cut back to approximately 5 cm at least twice prior to all trials, which commenced 2–3 weeks after cutting. Salinity treatment involved watering with 50 mM NaCl for 2 days, then 100 mM NaCl for 2 days, and 150 mM NaCl for the remainder of the trial (27 days). The control treatment consisted of plants being watered with regular water throughout. In all cases, volumetric soil moisture levels were maintained at approximately 60%, which was measured using a ML3 ThetaKit soil moisture meter (Hoskin Scientific Ltd., Burnaby, ON, USA) and has been found to be optimal for alfalfa under our growth conditions. With the exception of aboveground biomass assessments, all evaluations were carried out once salinity-treated plants were displaying stress-related symptoms (21–26 days following the initiation of treatment). In addition, pots were rotated daily to mitigate microclimate effects.

### 4.2. Assessment of Growth Characteristics

Ten biological replicate plants derived from vegetative stem cuttings were used to assess aboveground morphological characteristics under control and salinity treatments. Plant height was determined by measuring the length of the longest shoot; internode length comprised the mean value of the longest internode on the three longest shoots per plant; and the number of shoots consisted of the total number of primary, secondary, and tertiary shoots from each plant. Aboveground FW and DW were assessed once all plants had flowered. Aboveground FW was resolved by weighing all tissue above the crown immediately after harvest, and DW was established after drying at 65 °C for at least 1 week.

For root characteristics, 7 biological replicate plants derived from vegetative stem cuttings were evaluated under each growth condition. Root length assessments were carried out by removing plants from their pots, washing the roots thoroughly, and measuring the longest root on each plant. Root DW was established following drying at 65 °C for at least 1 week.

### 4.3. Measurement of Relative Water Content

Leaf RWCs were determined as described previously [83,141]. In brief, first, fully expanded trifoliate leaves from 10 biological replicate plants were harvested 21 days following the initiation of treatment (control and salinity). Fresh weights were determined immediately following harvest; turgid weights were established after submerging petioles in water in a closed microcentrifuge tube for 3–4 h; and DWs were recorded following the drying of turgid leaves at 80 °C overnight. RWC was calculated using the following equation: RWC (%) = [(FW − DW)/(TW − DW)] × 100.

### 4.4. Biochemical Assessments

For all biochemical assays, leaves were harvested 22 days following the initiation of treatment (control and salinity) and were then immediately flash frozen in liquid nitrogen and freeze-dried prior to carrying out assays. In all cases, absorbances were measured in microplate format using a Synergy Mx Multi-Mode Microplate Reader spectrophotometer (BioTek Instruments Inc., Winooski, VT, USA).

For the determination of MDA levels, first fully expanded trifoliate leaves from 8 biological replicate plants under each treatment, with 3 technical replicates per sample, were harvested and assessed using the QuantiChrom TBARS Assay Kit according to the manufacturer’s recommendations (BioAssay Systems, Hayward, CA, USA).

Proline assays were conducted as described previously [83], with minor modifications. In brief, 1 mL of 3% aqueous sulphosalicylic acid was added to approximately 10 mg of ground, freeze-dried, first fully expanded trifoliate leaf tissue from 10 biological replicates under each treatment, with 3 technical replicates for each. Reactions were incubated at room temperature for 3 h, and tubes were then centrifuged at 1500× *g* for 10 min. Either 600 µL of supernatant or L-proline dilutions for the generation of a standard curve were added to equal volumes of glacial acetic acid and ninhydrin reagent (0.025 g.mL^−1^ ninhydrin, 0.6 mL.mL^−1^ glacial acetic acid, 2.4 M H_3_PO_4_), and the resulting mixtures were incubated at 100 °C for 45 min. Reactions were then cooled on ice for 30 min, 1.2 mL toluene was added to each tube and vortexed, and mixtures were then centrifuged at 1000× *g* for 5 min. Proline content was determined using absorbances measured at 520 nm with toluene as a blank.

Total soluble carbohydrate contents were determined using 2 middle leaflets from first fully expanded trifoliate leaves from 5 biological replicate plants and the Plant Soluble Sugar Content Assay Kit (MyBioSource Inc., San Diego, CA, USA) in duplicate according to the manufacturer’s instructions.

### 4.5. Visualization of Reactive Oxygen Species

Fully expanded trifoliate leaves (third from the shoot tip) from 6 biological replicate plants under each treatment were harvested 21 days following the initiation of treatment and immersed in DAB staining solution (1 mg.mL^−1^ DAB in 10 mM Na_2_HPO_4_, pH 3.0) to detect H_2_O_2_ or in NBT staining solution (2 mg.mL^−1^ NBT in 50 mM sodium phosphate buffer, pH 7.5) to detect O_2_^−^. The leaf samples were vacuum infiltrated in solution for 5 min and then incubated overnight (DAB staining) or for 4 h (NBT staining) at 22 °C in the dark with shaking at 100 rpm. Chlorophyll was subsequently removed by boiling samples in 95% (*v*/*v*) ethanol for 20–30 min.

### 4.6. Determination of Total Antioxidant Capacity

Total antioxidant capacity was assessed using two middle leaflets from first fully expanded trifoliate leaves from 5 biological replicate plants under each treatment (salinity and control) 22 days after the initiation of treatment using the Total Antioxidant Capacity (T-AOC) Colorimetric Assay Kit following the manufacturer’s instructions (MyBioSource Inc., San Diego, CA, USA). Total protein contents were determined using the Bio-Rad Quickstart Protein Assay Kit (Bio-Rad Laboratories Inc., Hercules, CA, USA), and were used to calculate total antioxidant capacity (units.mg protein^−1^). Two technical replicates were used for each sample.

### 4.7. Assessment of Chlorophyll Levels and Photosynthetic-Related Parameters

Chlorophyll contents were assessed using three middle leaflets from third fully expanded trifoliate leaves per plant 22 days after the initiation of treatment, with 5 biological replicate plants evaluated under control and salinity treatments, respectively. Assessments were carried out using a CCM-200 chlorophyll content meter (Hoskin Scientific Ltd., Burlington, ON, USA).

A_sat_, g_sw_, E, ETR, C_i_, Φ_PSII_, and F_v_/F_m_ were determined using a LI-6800 (Li-Cor Inc., Lincoln, NE, USA) and 2 middle leaflets from first fully expanded trifoliate leaves from 5 biological replicate plants, respectively. Within the chamber, light intensity was maintained at 1500 µmol m^−2^ s^−1^, heat exchanger temperature was set at 24 °C, relative humidity at 50%, and CO_2_ concentration at 410 µmol CO_2_.mol^−1^ air. For A_sat_, g_sw_, E, and C_i_, leaves were assessed 22 days after the initiation of treatment, while ETR, Φ_PSII_, and F_v_/F_m_ were determined 25 days after the initiation of treatment. Leaves were dark-adapted prior to assessment of F_v_/F_m_ by wrapping leaflets in tin foil overnight. Each sample was stabilized within the chamber for 3 min prior to evaluation, and measurements were adjusted for leaf area where necessary, which was established using the Petiole Pro Plant Phenotyping leaf area meter app (version 1.6.2; https://play.google.com/store/apps/details?id=com.petiolepro.farmvision; accessed on 31 March 2023).

### 4.8. RNA-Seq Analysis

First fully expanded middle leaflets were harvested from 4 biological replicate control and saline-treated plants 25 days following the initiation of treatment, and were immediately flash frozen in liquid nitrogen and stored at −80 °C. Total RNA was extracted from each sample using the Spectrum Plant Total RNA Kit with on-column DNase treatment according to the manufacturer’s instructions (Sigma-Aldrich Corp., St. Louis, MO, USA), and the integrity of the resulting RNA was confirmed using a 2100 Bioanalyzer (Agilent Technologies, Santa Clara, CA, USA). Stranded mRNA libraries were generated using 250 ng of total RNA along with the NEBNext^®^ system (New England Biolabs Ltd., Whitby, ON, USA), and sequencing was conducted on an Illumina NovaSeq 6000 platform (Illumina Inc., San Diego, CA, USA) with 100-bp paired end reads by a service provider (Genome Québec Centre D’Expertise et de Services, Montreal, QC, Canada).

Raw reads were analyzed with the nf-core RNA-seq pipeline v3.0 [142,143], which utilizes Nextflow v21.02.0 [144]. In brief, raw reads were trimmed using Trim Galore v0.6.6 and read quality was determined using FastQC v0.11.9. High-quality filtered reads were mapped to the *Medicago truncatula* genome (Mt4.0 v2; https://plants.ensembl.org/Medicago_truncatula/; accessed on 28 July 2021), which is a close relative of alfalfa with a diploid and well-annotated genome, using the STAR aligner [145]. The quality of RNA-Seq data was assessed using RSeQC v3.0.1 [146], QualiMap v2.2.2 [147], and Picard v2.23.9. Transcripts were quantified using RSEM v1.3.1 [148], and expression levels of transcripts were normalized to counts per million using TMM normalization within edgeR [149].

Differential expression analysis was carried out in R version 4.0.3 using edgeR, and transcripts with an FDR below 0.05 were considered DEGs between treatments. PCA was also performed using R, while principal component scatter and volcano plots were generated in R using ggplot2 v3.4.1 [150] and cowplot v1.1.1 [151] packages. GO term enrichment analysis of up- and down-regulated genes was carried out separately using the Singular Enrichment Analysis (SEA) tool in AgriGO v2 (http://systemsbiology.cau.edu.cn/agriGOv2/; accessed on 16 June 2022) [152] with *M. truncatula* locus ID v4.0 (JCVI) as the reference species, along with the hypergeometric statistical test method, Yekutieli (FDR under dependency) as the multi-test adjustment method, and a significance level of 0.05.

The determination of significant DEG-associated pathways between control and saline conditions was achieved using MapMan v3.6 (https://mapman.gabipd.org/; accessed on 25 March 2022) with the *M. truncatula* genome as a reference (Mt4.0 v2). The sequence data generated in this study are available at the National Center for Biotechnology Information (NCBI) Sequence Read Archive (BioProject ID PRJNA952537).

### 4.9. Validation of RNA-Seq Results Using Quantitative RT-PCR (qRT-PCR)

The same total RNA that was utilized for RNA-Seq analysis was used for qRT-PCR validation. First-strand cDNA synthesis was conducted using the SuperScript VILO cDNA synthesis kit (Thermo Fisher Scientific, Waltham, MA, USA) with 500 ng of total DNase-treated RNA. Quantitative RT-PCR assays were carried out in a final reaction volume of 10 µL using an appropriate dilution of cDNA template from each sample, PerfeCTa SYBR Green Supermix (Quantabio, Beverly, MA, USA), and primers designed to anneal to the coding regions of 8 genes that were selected based on their differential expression in the RNA-Seq experiment (see Table S2 for primer sequences). A 127-nt and 69-nt region of the constitutively expressed *GLYCERALDEHYDE 3-PHOSPHATE DEHYDROGENASE* (*G3PD*) and *Msc27* genes, respectively, which have both been demonstrated previously to serve as a suitable reference gene for qRT-PCR in alfalfa under salinity and control conditions [23,153], were utilized as internal controls. A Quantstudio 6 Flex Real-Time PCR system (Thermo Fisher Scientific) was used to carry out the assays. Thermal parameters included an initial denaturation at 95 °C for 3 min, followed by 40 cycles of 95 °C for 15 s and 60 °C for 45 s. Dissociation curves were generated to confirm that only a single amplification product was generated in each case. Gene expression levels were determined using the ΔΔCt method and Applied Biosystems^TM^ analysis software v4.0 (Thermo Fisher Scientific), with expression levels comprising the mean values of 4 biological replicates (3 technical replicates of each) normalized to those of the internal controls. Log_2_ fold-change values between control and saline-treated samples were then calculated and compared to RNA-Seq values for the determination of correlation coefficients.

### 4.10. Metabolomic Analysis

First fully expanded trifoliate leaves from 7 biological replicate plants grown under each growth condition, respectively, were harvested 26 days after the initiation of salinity treatment, immediately frozen in liquid nitrogen, and stored at −80 °C. Approximately 10 mg of each sample were homogenized in 1 mL of 90% ice-cold methanol in water with glass beads using a bead beater (Mini-beadbeater-16, BioSpec, Bartlesville, OK, USA). Following homogenization, beads were rinsed twice with 200 µL of 90% methanol, and the homogenate and rinses were combined in single tubes. Samples were placed at −20 °C overnight to allow for complete protein precipitation, after which time they were centrifuged at 14,000 rpm for 15 min at 4 °C, and the clear supernatant was transferred into a new tube and dried. Samples were reconstituted in water/acetonitrile (1:1, *v*/*v*), centrifuged at 14,000 rpm for 15 min at 4 °C, and the clear supernatant was used for liquid chromatography-mass spectrometry (LC-MS) analysis.

LC-MS analysis was performed using an Agilent 1290 Infinity II UHPLC System (Agilent Technologies) coupled with an ultra-high-resolution Qq-time-of-flight (UHR-QqTOF) mass spectrometer (Impact II; Bruker Corp., Billerica, MA, USA). For electrospray ionization negative (ESI-) mode analysis, hydrophilic interaction liquid chromatography (HILIC) separation was performed using a SeQuant ZIC^®^-pHILIC column (150 × 2.1 mm, 5 μm, 200 Å) (EMD Millipore, Burlington, MA) maintained at 35 °C. Mobile phase A consisted of water/acetonitrile (95:5, *v*/*v*) with 10 mM ammonium acetate (pH adjusted to 9.8 using ammonium hydroxide), and mobile phase B consisted of water/acetonitrile (5:95, *v*/*v*). The LC gradient was set as follows: 0 min, 95% B; 20 min, 5% B; 21 min, 95% B; 35 min: 95% B. The flow rate was 0.15 mL.min^−1^. The injection volumes were optimized by quality control (QC) samples and set at 2.0 μL. To correct MS signal intensities, a series of QC samples were injected with five different volumes: 0.5, 1.0, 2.0, 3.0, and 4.0 µL. Each serial QC sample had two technical replicates. The MS settings were as follows: capillary voltage, 3600 V; dry gas temperature, 220 °C; dry gas, 7.0 L.min^−1^; nebulizer gas pressure, 1.6 bar; mass scan range, 70–1500 (*m*/*z*); spectra rate, 8.00 Hz; and cycle time, 2.0 s. For centroid spectra calculation, the peak summation width was 3 pts. The mass spectrometer was calibrated using sodium formate to improve mass accuracy.

For electrospray ionization positive (ESI+) mode analysis, RP liquid chromatography separation was performed using a Waters reversed phase UPLC Acquity BEH C18 Column (1.7 μm, 1.0 mm × 100 mm, 130 Å) (Milford, MA, USA) maintained at 35 °C. Mobile phase A consisted of water, and mobile phase B consisted of acetonitrile. Both contained 0.1% formic acid. The LC gradient was set as follows: 0 min, 5% B; 8 min, 25% B; 14 min, 70% B; 20 min, 95% B; 23 min, 95% B; 23.01 min, 5% B; 30 min, 5% B. The flow rate was 0.15 mL.min^−1^. The injection volumes were optimized using QC samples and were ultimately set at 4.0 μL. To correct MS signal intensities, a series of QC samples were injected with five different volumes: 1.0, 2.0, 4.0, 6.0, and 8.0 µL. Each serial QC sample had two technical replicates. The MS settings were as follows: capillary voltage, 4500 V; dry gas temperature, 220 °C; dry gas, 7.0 L.min^−1^; nebulizer gas pressure, 1.6 bar; mass scan range, 70–1500 (*m*/*z*); spectra rate, 8.00 Hz; and cycle time, 2.0 s. For centroid spectra calculation, the peak summation width was 3 pts. The mass spectrometer was calibrated using sodium formate to improve mass accuracy.

Raw LC-MS data files were first converted to ABF format using Reifycs Abf Converter ver. 4.0.0 (https://www.reifycs.com/AbfConverter/; accessed on 5 April 2023) and were then processed in MS-DIAL ver. 4.80 [154] for peak detection, peak alignment, and putative metabolic annotation. Detailed processing parameters can be found in File S5. The resulting aligned metabolic feature table from MS-DIAL was further processed using the MAFFIN package [155] and then exported for comparative analysis. Metabolite annotation was performed using the default MS-DIAL library combined with the National Institute of Standards and Technology 2020 mass spectral library, and confirmation was carried out by comparing against metabolic standards. F-tests were performed to assess the equality of variance in the two groups, and *t*-tests were then performed to determine whether metabolic features differed significantly between treatment groups. F-tests and *t*-tests were performed using Excel. PCA was conducted using MetaboAnalyst 5.0 [156] to evaluate the specificity of the metabolic profiles of the two groups. The intensity of metabolic features was subjected to square root transformation and auto-scaling. Metabolites were classified using the Human Metabolome Database (HMDB; https://hmdb.ca/metabolites; accessed on 31 March 2023).

### 4.11. Joint Pathway Analysis

Joint-pathway analysis was performed using lists of significantly altered metabolites and transcripts along with Metaboanalyst 5.0 [156] to investigate metabolic pathway alterations in salinity-treated compared to control-treated plants. For joint-pathway analysis, *M. truncatula* gene IDs for significant DEGs identified in our RNA-Seq analysis were converted to *Arabidopsis thaliana* gene IDs using the orthology search function in g:Profiler [157], whereby 68% of *M. truncatula* DEGs possessed an Arabidopsis ortholog (File S1). Each *M. truncatula* gene ID was limited to a single corresponding Arabidopsis gene ID, which was used in joint pathway analysis along with those metabolites with HMDB IDs. Parameters were set to assess all pathways (integrated), the hypergeometric test for enrichment analysis, degree centrality for measurement of topology, and integration by combining *p*-values (pathway level). Pathways were considered enriched when the FDR was below 0.05.

### 4.12. Statistical Analysis

With respect to morphological, physiological, and biochemical assessments, statistical distinctions between the means of salt- and control-treated alfalfa plants were resolved using 2-tailed student’s *t*-tests assuming unequal variance. Means were deemed significantly different at *p* ≤ 0.05. The statistical treatment of RNA-Seq and metabolomic data is described within their associated sections.

## Data Availability

RNA-Seq data generated in this study are available at the National Center for Biotechnology Information (NCBI) Sequence Read Archive (BioProject ID PRJNA952537).

## Supplementary Materials

The following supporting information can be downloaded at: https://www.mdpi.com/article/10.3390/plants12102059/s1, Figure S1: Root length and dry weight under control and saline conditions; Figure S2: Overview of RNA-Seq analysis of alfalfa leaves under control and salinity conditions; Figure S3: Correlation between RNA-Seq and qRT-PCR data in the leaves of alfalfa cv. Beaver under salinity vs. non-saline conditions; Figure S4: Overview of metabolomic analysis of alfalfa leaves under control and salinity conditions; File S1: List of DEGs and annotations; File S2: List of MapMan bins for DEGs of interest; File S3: List of significantly altered metabolites and classification data; File S4: List of matched features from joint pathway analysis; File S5: Details of metabolomic data processing methods; Table S1: RNA-Seq read alignment data for alfalfa cv. Beaver leaf tissue under control and saline conditions; Table S2: Primers used for qRT-PCR validation of RNA-Seq results.
