# Supplementary material for: Elucidation of Physiological, Transcriptomic and Metabolomic Salinity Response Mechanisms in Medicago sativa
Source: Plants (Basel). 2023 May 22;12(10):2059. doi: 10.3390/plants12102059 (PMC10221938; doi:10.3390/plants12102059)
Supplement: Supplementary file 1 [file plants-12-02059-s001.zip › plants-2403550-supplementary/Supplemental Figure 1 Root morphology (May 31 2023).pdf]

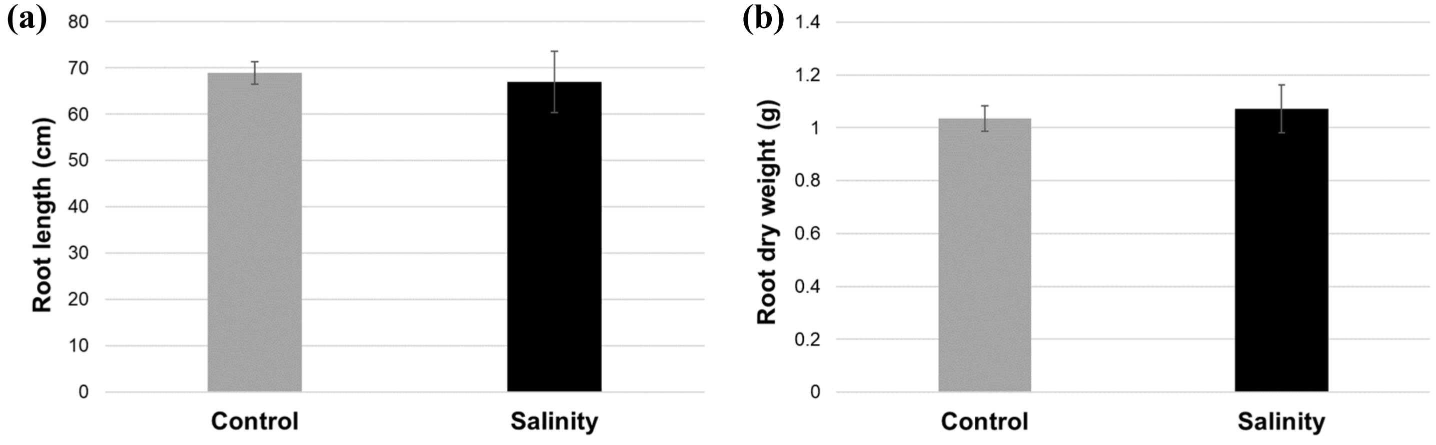

Supplemental Figure S1. Root length and dry weight under control and saline conditions. Measurements were taken 26 days following the initiation of salinity treatment and 43 days following cutting. Blocks denote mean values of 7 biological replicate plants derived from stem cuttings under each treatment, and bars represent standard errors.
