# Supplementary material for: Elucidation of Physiological, Transcriptomic and Metabolomic Salinity Response Mechanisms in Medicago sativa
Source: Plants (Basel). 2023 May 22;12(10):2059. doi: 10.3390/plants12102059 (PMC10221938; doi:10.3390/plants12102059)
Supplement: Supplementary file 1 [file plants-12-02059-s001.zip › plants-2403550-supplementary/Supplemental Figure 2 Volcano and PCA RNASeq (Mar 31 2023).pdf]

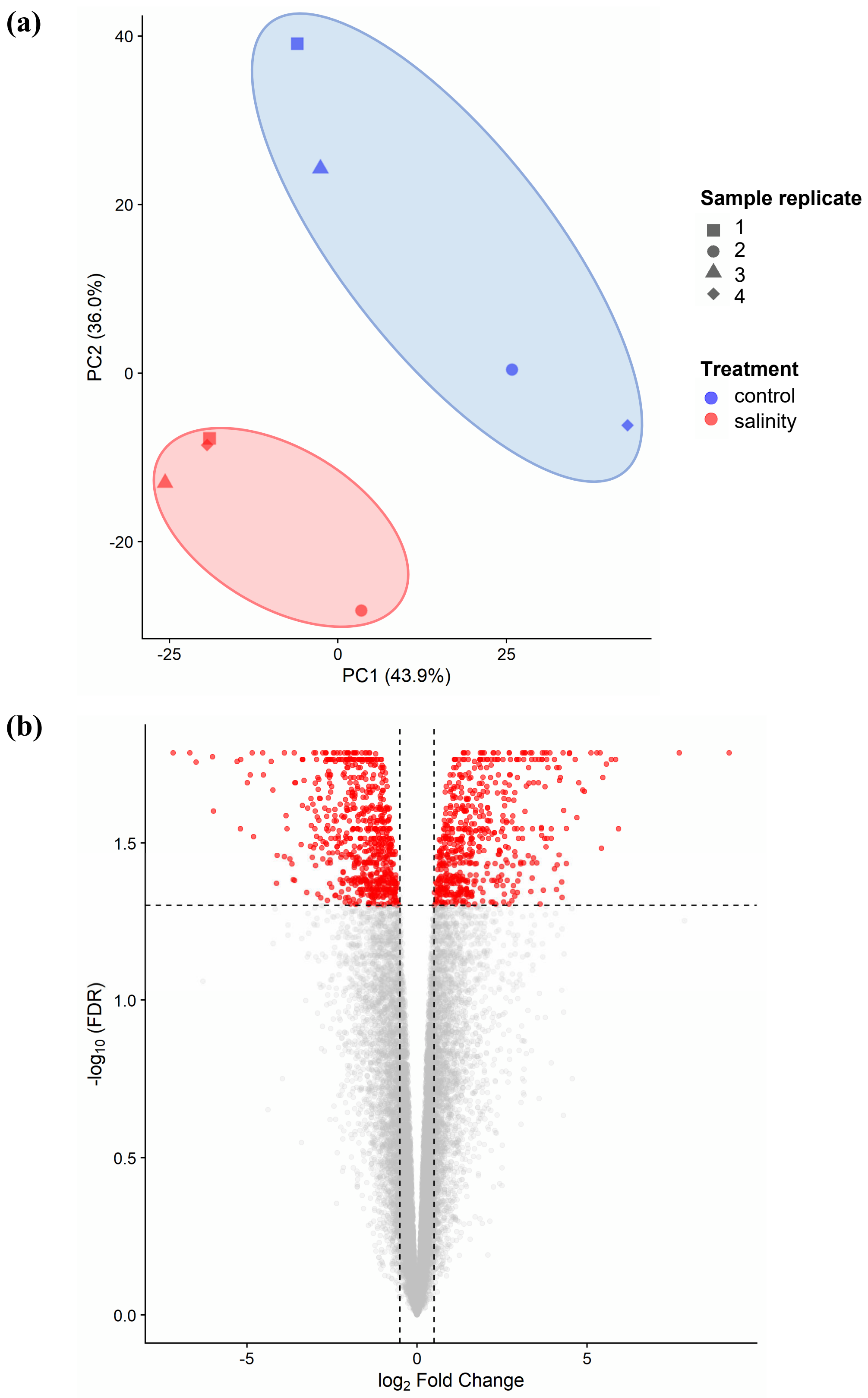

Supplemental Figure S2. Overview of RNA-Seq analysis of alfalfa leaves under control and salinity conditions. **(a)** Principal component analysis of RNA-Seq data. Leaf RNA from four biological replicates were assessed under each treatment (control and salinity). **(b)** Volcano plot of differentially expressed genes between control and salinity conditions. The x-axis indicates log_2 fold-change values and the y-axis indicates the statistical significance of the degree of change. Red points denote DEGs above the selected threshold for fold-change and significance. FDR, false discovery rate.
