# Supplementary material for: Elucidation of Physiological, Transcriptomic and Metabolomic Salinity Response Mechanisms in Medicago sativa
Source: Plants (Basel). 2023 May 22;12(10):2059. doi: 10.3390/plants12102059 (PMC10221938; doi:10.3390/plants12102059)
Supplement: Supplementary file 1 [file plants-12-02059-s001.zip › plants-2403550-supplementary/Supplemental Figure 3 qRT-PCR validation (March 31 2023).pdf]

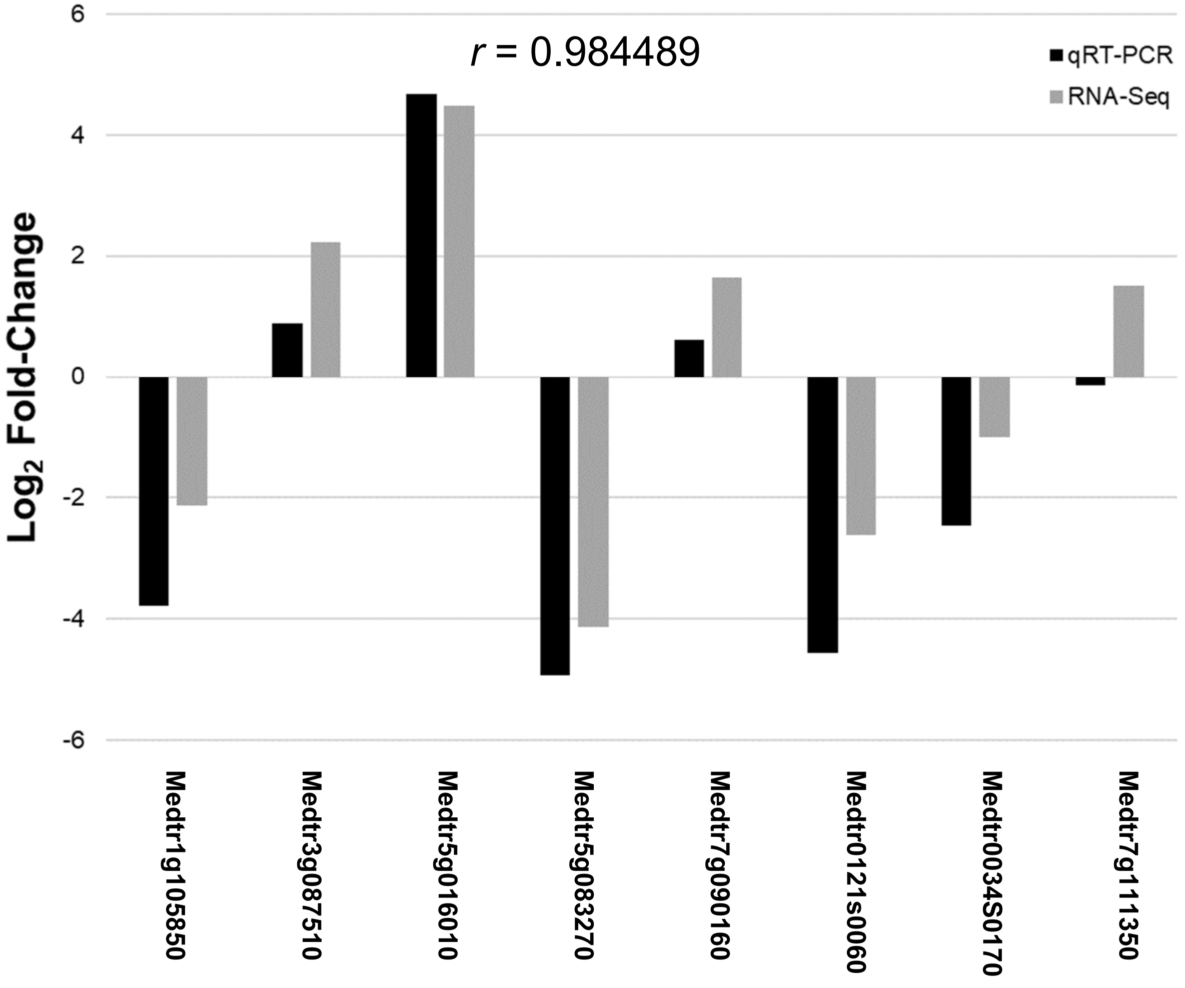

Supplemental Figure S3. Correlation between RNA-Seq and qRT-PCR data in the leaves of alfalfa cv. Beaver under salinity vs. non-saline conditions. Bars denote log_2-fold changes between control and salinity conditions with qRT-PCR values displayed in black and RNA-Seq values shown in gray. *r* indicates the correlation coefficient between qRT-PCR and RNA-Seq log_2 fold-changes.
