# Supplementary material for: Elucidation of Physiological, Transcriptomic and Metabolomic Salinity Response Mechanisms in Medicago sativa
Source: Plants (Basel). 2023 May 22;12(10):2059. doi: 10.3390/plants12102059 (PMC10221938; doi:10.3390/plants12102059)
Supplement: Supplementary file 1 [file plants-12-02059-s001.zip › plants-2403550-supplementary/Supplemental Figure 4 Metabolomics (March 31 2023).pdf]

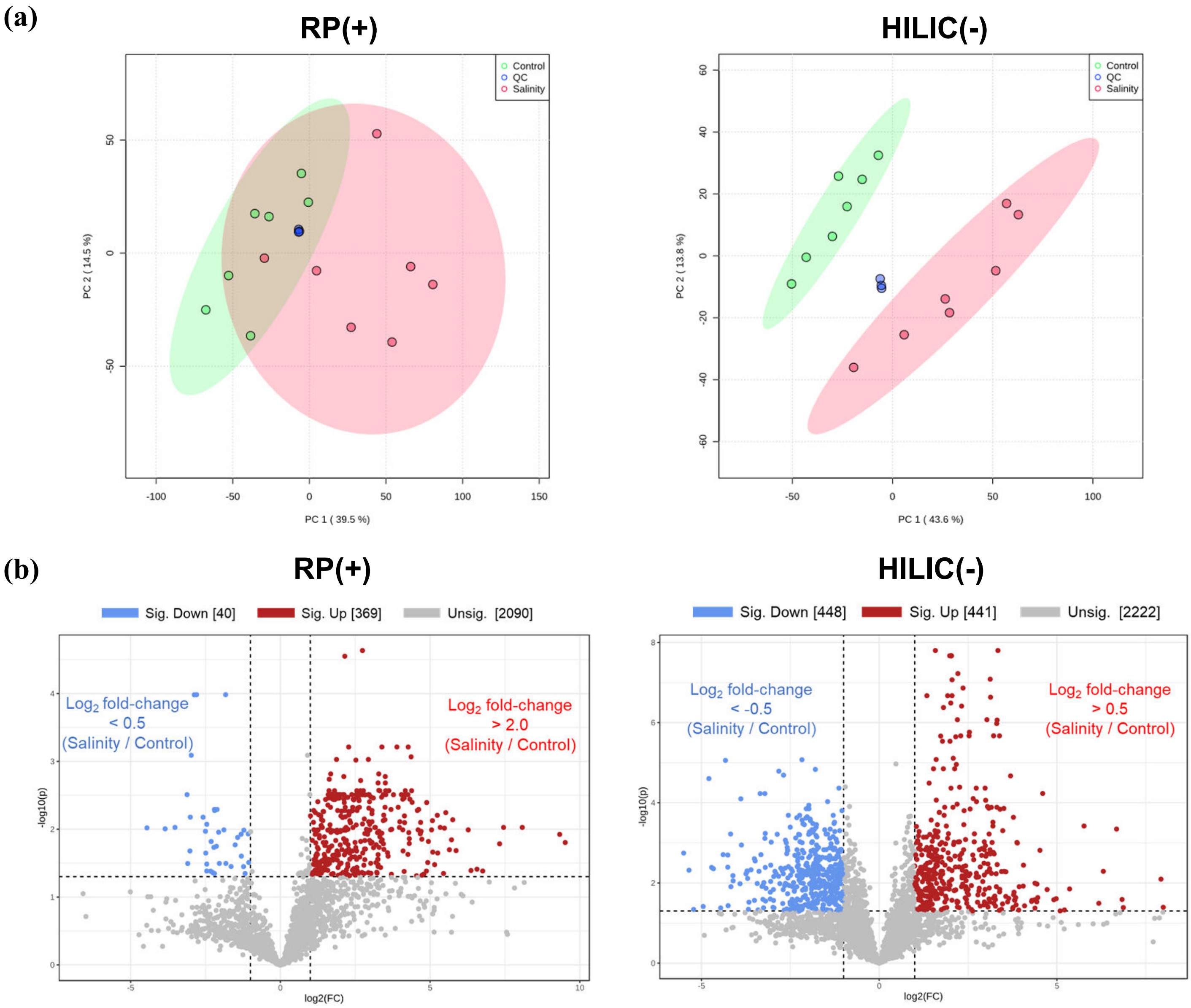

Supplemental Figure S4. Overview of metabolomic analysis of alfalfa leaves under control and salinity conditions. **(a)** Principal component analysis of metabolomic data. **(b)** Volcano plot of significant differentially abundant metabolites between control and salinity conditions. The x-axis indicates $\log_2$ fold-change values and the y-axis denotes the statistical significance of the degree of change in RP (+) and HILIC (-).
