# Supplementary material for: Elucidation of Physiological, Transcriptomic and Metabolomic Salinity Response Mechanisms in Medicago sativa
Source: Plants (Basel). 2023 May 22;12(10):2059. doi: 10.3390/plants12102059 (PMC10221938; doi:10.3390/plants12102059)
Supplement: Supplementary file 1 [file plants-12-02059-s001.zip › plants-2403550-supplementary/Supplemental File 5 Metabolomic processing (March 28 2023).pdf]

Metabolomic data processing

MS-DIAL parameter settings for HILIC (-) data processing
MS-DIAL parameters were optimized by *Paramounter*[3] as follows:
Software version
MS-DIAL version 4.90

#Project
MS1 Data type: Centroid
MS2 Data type: Centroid
Ion mode: Negative
Target: Metablomics
Mode: ddMSMS
#Data collection parameters
Retention time begin: 0
Retention time end: 100
Mass range begin: 0
Mass range end: 2000
MS2 mass range begin: 0
MS2 mass range end: 2000
#Isotope recognition
Maximum charged number: 2
Mass accuracy
MS^1 tolerance: 0.01 Da, MS^2 tolerance: 0.025 Da
Peak detection
Minimum peak height: 364
Mass slice width: 0.01 Da
Smoothing method: Linear weighted moving average
Smoothing level: 3 scan
Minimum peak width: 3 scan
Identification
Accurate mass tolerance (MS^1): 0.01 Da
Accurate mass tolerance (MS^2): 0.05 Da
Identification score cut off: 70%
The retention time is not used for scoring.
Library
MS/MS reference library (version 15) provided by MS-DIAl, combined with NIST 20 library
Adduct
$[M-H]^-$
Alignment
Retention time tolerance: 0.105 min
MS^1 tolerance: 0.007 Da

MS-DIAL parameter settings for RP (+) data processing, optimized by *Paramounter* as follows:

Software version

MS-DIAL version 4.80

#Project

MS1 Data type: Centroid

MS2 Data type: Centroid

Ion mode: Positive

Target: Metablomics

Mode: ddMSMS

#Data collection parameters

Retention time begin: 0

Retention time end: 100

Mass range begin: 0

Mass range end: 2000

MS2 mass range begin: 0

MS2 mass range end: 2000

#Isotope recognition

Maximum charged number: 2

Mass accuracy

MS^1 tolerance: 0.02 Da, MS^2 tolerance: 0.025 Da

Peak detection

Minimum peak height: 442

Mass slice width: 0.02 Da

Smoothing method: Linear weighted moving average

Smoothing level: 3 scan

Minimum peak width: 3 scan

Identification

Accurate mass tolerance (MS^1): 0.01 Da

Accurate mass tolerance (MS^2): 0.05 Da

Identification score cut off: 70%

The retention time is not used for scoring.

Library

MS/MS reference library (version 15) provided by MS-DIAl, combined with NIST 20 library

Adduct

$[M+H]^+$, $[M+NH_4]^+$, $[M+Na]^+$

Alignment

Retention time tolerance: 0.154 min

MS^1 tolerance: 0.009 Da

MAFFIN processing setting

The processing workflow of MAFFIN refers to https://github.com/HuanLab/MAFFIN and https://doi.org/10.1093/bioinformatics/btac355.

All parameters were set as default except for the blank filter cut-off, which was set as 5 for both HILIC(-) and RP(+).
