# Supplementary material for: Elucidation of Physiological, Transcriptomic and Metabolomic Salinity Response Mechanisms in Medicago sativa
Source: Plants (Basel). 2023 May 22;12(10):2059. doi: 10.3390/plants12102059 (PMC10221938; doi:10.3390/plants12102059)
Supplement: Supplementary file 1 [file plants-12-02059-s001.zip › plants-2403550-supplementary/Supplemental Tables (May 1 2023).pdf]

Table S1. RNA-Seq read and alignment data for alfalfa cv. Beaver leaf tissue under control and saline conditions

	# Raw Reads	# Mapped Reads	# of Expressed Transcripts
C-1	193,486,090	142,603,507	19,168
C-2	231,379,036	160,167,048	19,216
C-3	133,009,552	96,692,166	19,181
C-4	170,006,848	116,226,568	19,170
S-1	166,539,732	119,238,137	19,223
S-2	187,496,544	131,445,620	19,222
S-3	135,077,072	97,396,305	19,210
S-4	126,016,842	90,052,470	19,210

C, control conditions (non-saline); S, saline conditions.

Table S2. Primers used for qRT-PCR validation of RNA-Seq results

Gene ID	Description	Primer sequences (5' – 3')	Amplicon length (bp)
Medtr1g105850	Salt stress response/antifungal domain protein	F: ACAACCTCCATCACCAATACC<br>R: CAACGGGATCATATCCACTTCA	97
Medtr3g087510	PLAT-plant-stress protein	F: GTCCTCATCATGGGTGGTATTG<br>R: GCAAGCCATTGTTCCACTTC	100
Medtr5g016010	Peroxidase family protein	F: TGCCACTCTACGTCTCTTCTT<br>R: GGTGATCCCTCTCTGCTCTATT	101
Medtr5g083270	Abscisic acid receptor	F: TTCGACTTGTTTCCGGTCTAC<br>R: CACCACCAACAACACTGAAAC	99
Medtr7g090160	Pyrroline-5-carboxylate reductase	F: GTCGTCGTCTTCTCGGTTAAA<br>R: GATTCCAGCAGCAACAGAAAC	105
Medtr0121s0060	Stress responsive A/B barrel domain protein	F: CCCTCACATTCTCACCATTCA<br>R: CTTGGGAAGTAGAGGACATCTTG	117
Medtr0034s0170	Trehalose-6-phosphate synthase	F: GAGCCGATCTGTTGAAGTTAGA<br>R: GGTGATGTCATGGATTTGCTATG	99
Medtr7g111350	Low temperature and salt responsive family protein	F: GCACAGCTACCTTCGTTGATA<br>R: TCCAGAACTCCACTTCCAAAC	90
G3PD*	Glyceraldehyde-3-phosphate dehydrogenase	F : CATCACAGCCACTCAGAAGAC<br>R: TGGAAGCACTTTGCCTACAG	127
Msc27**	Translationally-controlled tumor-like protein	F: GTTGAAGTAGACATTGGTGCTAACG<br>R: AGCTGAGTCATCAACACCCTCA	69

* Primer sequences obtained from Kaundal et al. 2021 [23].

** Primer sequences obtained from Wang et al. 2015 [152].
